# Homozygous CHD8 mutation intensifies ASD phenotypes and attenuates sex differences

**DOI:** 10.1038/s41380-026-03646-9

**Published:** 2026-05-09

**Authors:** Jinkyeong Kim, Seungjoon Lee, Eunkyu Hwang, Hwajin Jung, Chanhee Lee, Sang-Han Choi, Sooyeon Lee, Seongbin Kim, Heera Moon, Jisoo Kim, Gina Lee, Yong Gyu Kim, Soogeun Shin, Hyojin Kang, Se Jin Kim, Heon Yung Gee, Seong-Gi Kim, Eunee Lee, Eunjoon Kim

**Affiliations:** 1 Department of Biological Sciences, Korea Advanced Institute of Science and Technology (KAIST), Daejeon, Korea; 2 Center for Synaptic Brain Dysfunctions, Institute for Basic Science (IBS), Daejeon, Korea; 3 Department of Anatomy, Graduate School of Medical Science, Yonsei University College of Medicine, Brain Korea 21 Project, Seoul, Korea; 4 Center for Neuroscience Imaging Research, Institute for Basic Science (IBS), Suwon, Korea; 5 Department of Biomedical Engineering, Sungkyunkwan University, Suwon, Korea; 6 Division of National Supercomputing, Korea Institute of Science and Technology Information (KISTI), Daejeon, Korea; 7 Department of Pharmacology, Graduate School of Medical Science, Yonsei University College of Medicine, Brain Korea 21 Project, Seoul, Korea

**Keywords:** Neuroscience, Genetics

## Abstract

CHD8 is a chromatin remodeler implicated in autism spectrum disorders (ASD) and multiple neurodevelopmental disorders, yet heterozygous Chd8-mutant mouse lines often exhibit only mild ASD-related phenotypes, leaving its role unclear. Because a complete knockout of Chd8 causes embryonic lethality, we generated viable homozygous Chd8-mutant mice carrying the human CHD8-Asn2373LysfsX2 mutation using a hybrid (C57BL6/J × 129/Sv) genetic background. Compared to heterozygous *Chd8*^*+/N2373K*^ mice, the homozygous *Chd8*^*N2373K/N2373K*^ mice showed more robust phenotypes, including increased ASD-related behaviors and brain volume, decreased cerebral blood volume/flow, brain rhythms, and synaptic transmission, and ASD-related transcriptomic changes. Notably, while *Chd8*^*+/N2373K*^ mice on a pure background predominantly displayed behavioral deficits in males, the homozygous mutants in the hybrid background exhibited more pronounced female phenotypes, suggesting the interaction of genetic background and mutation strength. A direct comparison of *Chd8*^*+/N2373K*^ and *Chd8*^*N2373K/N2373K*^ mice on the same hybrid background across brain volume, cerebral blood flow, neuronal firing, synaptic transmission, and transcriptome revealed a gene dosage-dependent attenuation of sexual dimorphic phenotypes that varied by developmental stage and brain region. Transcriptomic analyses further implicated pathways related to synaptic function, RNA splicing, and mitochondrial activity in mediating differences in male–female protection and susceptibility. Thus, a homozygous Chd8 mutation not only intensifies ASD-related traits but can also diminish typical sex-specific severity patterns, uncovering a novel link between mutation strength and sexual dimorphism in ASD.

## Introduction

*CHD8*, encoding a chromatin remodeler, is implicated in approximately 0.2–0.4% of ASD cases [1–7] as well as in other neurodevelopmental disorders such as intellectual disability, developmental delay, ADHD, neurological problems, sleep disturbances, and schizophrenia [8, 9]. CHD8 influences many other ASD-risk genes and is critical for neural development and function [10], as demonstrated by studies, including those using *Chd8*-mutant mice [10–48]. While murine models recapitulate aspects of CHD8-related pathology (e.g., macrocephaly), behavioral phenotypes often fail to robustly mirror core ASD-like phenotypes (e.g., repetitive behavior), complicating mechanistic investigations [11–13, 17, 19, 21, 32, 45]. This is partly because homozygous *Chd8* mutation usually leads to embryonic lethality in mice, although hypomorphic *Chd8* mutants have been reported to display gene deletion dosage-dependent variations in transcriptomic and brain size phenotypes [31].

We previously reported heterozygous *Chd8*-mutant mice carrying the patient-derived knock-in mutation Asn2373LysfsX2 (*Chd8* ^*+/N2373K*^ mice) [19, 49]. These mice exhibit male-preponderant molecular, synaptic, neuronal, and behavioral phenotypes reminiscent of ASD [19], similar to the results from human genetics [4, 50] and other *Chd8*-mutant mice [35], although homozygous mutant (*Chd8* ^*N2373K/N2373K*^) mice were embryonically lethal, preventing us from observing stronger behavioral phenotypes and exploring underlying mechanisms [19]. Notably, female *Chd8* ^*+/N2373K*^ mice appeared to possess protective neurobiological features such as reduced baseline neuronal excitability and elevated inhibitory synaptic transmission and inhibitory neuronal firing [19] that may mitigate ASD-like impairments, rendering the phenotypes in male *Chd8* ^*+/N2373K*^ mice more pronounced. If these female protective effects are correct, then a stronger *Chd8* mutation might overpower them, resulting in deficits in both male and female mutants and providing direct evidence for gene dosage–dependent suppression of sexually dimorphic phenotypes in ASD. Producing viable *Chd8*^*N2373K/N2373K*^ mice would allow us to address these two key research aims.

To overcome embryonic lethality, we switched the genetic background of *Chd8*^*N2373K/N2373K*^ mice from pure C57BL6/J to a hybrid (C57BL6/J × 129/Sv) background, which rescued the viability of homozygous mutants. On this hybrid background, *Chd8*^*N2373K/N2373K*^ mice displayed more severe behavioral deficits compared to *Chd8* ^*+/N2373K*^ mice, with these deficits being notably more pronounced in females, a stark contrast to the patterns observed on the pure background. Comprehensive assessments, including brain volume (MRI), cerebral blood volume/flow (fMRI), neuronal firing, synaptic function, and transcriptomic profiling—revealed a gene dosage–dependent intensification of phenotypes alongside suppressed and, in specific molecular readouts, partially reconfigured sexual dimorphism.

## Results

### Generation of viable homozygous *Chd8*^*N2373K/N2373K*^ mice

**Fig. 1:**
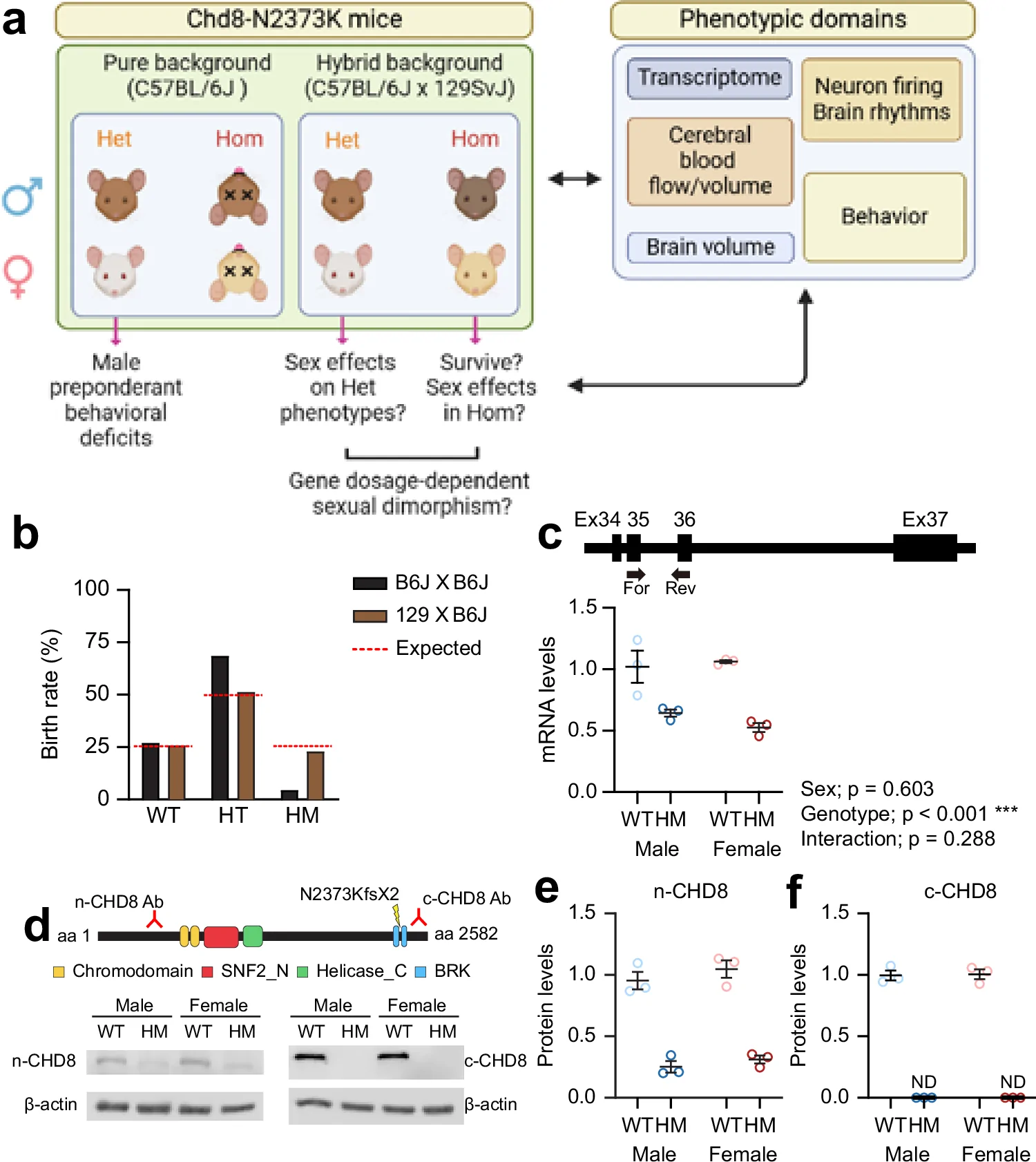
Generation of viable homozygous *Chd8*^*N2373K/N2373K*^ mice. **a** Experimental design testing the FPE hypothesis using a *Chd8*-mutant (CHD8-N2372K) mouse model of ASD and manipulating the genetic dosage (heterozygous/Het and homozygous/Hom) and genetic background. In a pure genetic background (C57BL/6 J), homozygous mutant mice are lethal. This lethality may be overcome by shifting the genetic background from a pure (C57BL6J) to hybrid (C57BL6J x 129/Sv) background in which we can compare various phenotypes (behavior, anatomical brain volume, functional cerebral blood flow/volume, neuronal firing, synaptic transmission, and transcriptome) from heterozygous and homozygous males and females. These data would enable to assess if a homozygous Chd8 mutation can induce core ASD-like behaviors (i.e., self-grooming) and affects sexual dimorphic phenotypes in a gene deletion-dosage dependent manner. **b** Mendelian ratios of wild-type (WT), *Chd8*^*+/N2373K*^ (HT), and *Chd8*^*N2373K/N2373K*^ (HM) mice (~P7) in the pure (C57BL6/J) and hybrid (C57BL6/J x 129/Sv) genetic backgrounds. Note that the hybrid genetic background normalizes the Mendelian ratio. **c** Decreased levels of *Chd8* mRNAs (~50–60% of WT levels) in the brains of *Chd8*^*N2373K/N2373K*^ (HM) male and female mice (~P21), suggestive of nonsense-mediated decay, as determined by RT-qPCR. For/Rev indicates forward/reverse primers. **d**–**f** Decreased levels of CHD8 proteins (~20% of WT levels) in the brain of *Chd8*^*N2373K/N2373K*^ (HM) male and female mice (~2–3 months), as determined by immunoblot analysis of whole-brain lysates with antibodies targeting the N- and C-terminal regions of the protein, the positions of which are indicated above the protein domain diagram. Note that the C-terminal portion of the protein (~200 aa after the truncation point) is completely removed in *Chd8*^*N2373K/N2373K*^ mice, while the N-terminal portion of the protein before the truncation point is decreased to ~20% of WT levels, indicative of truncation-induced protein destabilization. SNF2_N, SNF2 family N-terminal domain; Helicase_C, Helicase conserved C-terminal domain; BRK, Brahma and Kismet domain.

Our previous study using *Chd8*-mutant mice with a human CHD8 frameshift mutation (N2373KfsX) [49] showed male-preponderant phenotypes in heterozygous (*Chd8*^*+/N2373K*^) mice [19]. Investigating homozygous (*Chd8*^*N2373K/N2373K*^) mice could reveal stronger core ASD-related traits, such as increased self-grooming, and help assess how mutation strength affects sexual dimorphism. However, embryonic lethality has hindered this line of research. To address this limitation, we switched the genetic background from pure C57BL6/J to a hybrid C57BL6/J × 129/Sv (Fig. 1a), a strategy known to mitigate lethality through enhanced genetic diversity and buffering of deleterious mutations [51].

This change markedly suppressed the embryonic lethality and normalized the Mendelian ratios of wild-type (WT), *Chd8*^*+/N2373K*^, and *Chd8*^*N2373K/N2373K*^ mice (Fig. 1b). *Chd8* mRNAs in hybrid-background *Chd8*^*N2373K/N2373K*^ mice were reduced to ~40–50% of WT levels (Fig. 1c), suggestive of nonsense-mediated decay. CHD8 proteins in *Chd8*^*N2373K/N2373K*^ mice were decreased to ~20% of WT levels, as shown by immunoblot results using N- and C-terminal-targeting antibodies and whole-brain and brain region-specific lysates (Fig. 1d–f; Supplementary Fig. 1), suggesting that the truncated CHD8 proteins lacking the last ~200 aa residues are unstable, and their levels are reduced to ~20% of WT levels.

### Sexually dimorphic behavioral deficits in *Chd8*^*N2373K/N2373K*^ mice

**Fig. 2:**
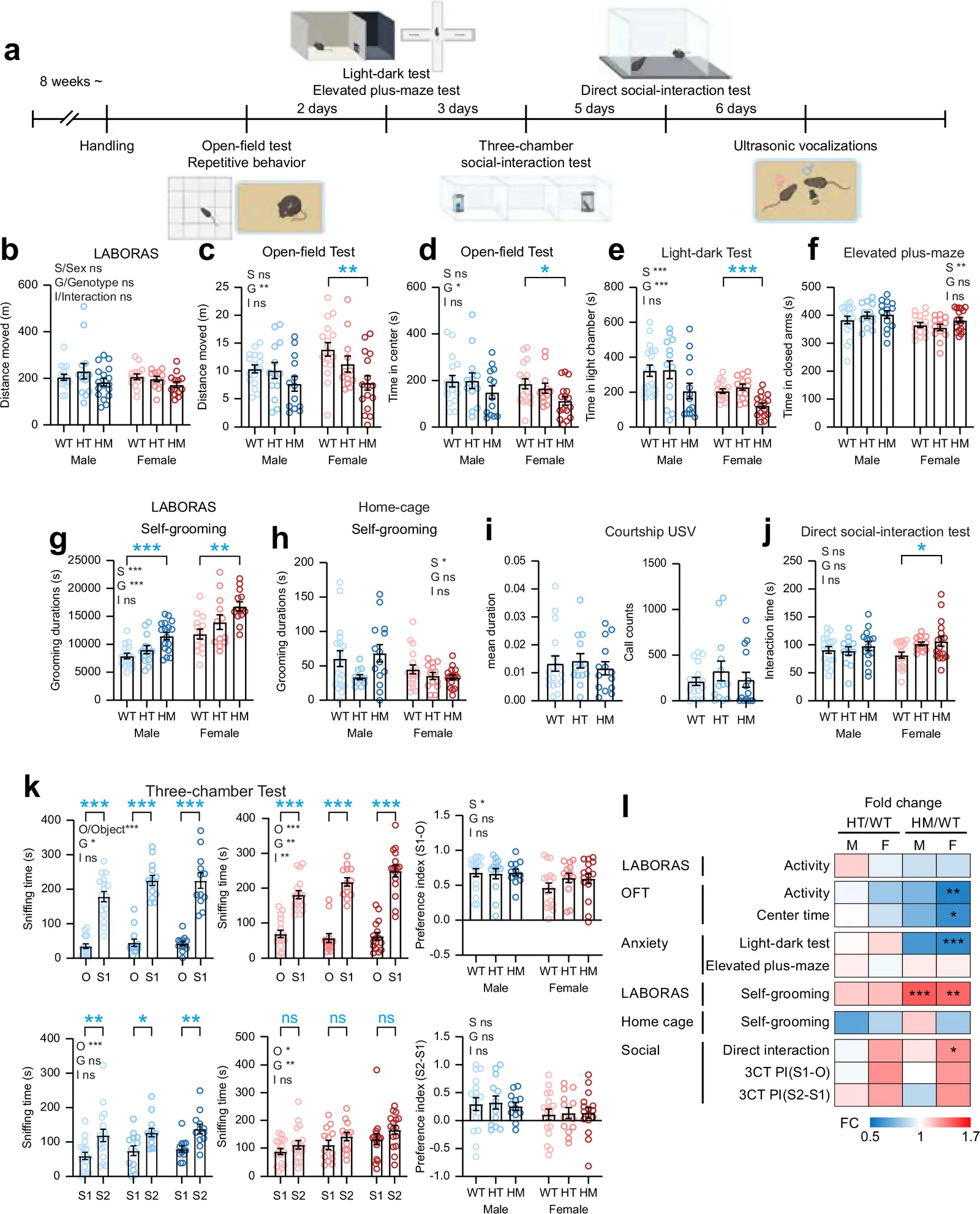
Sexually dimorphic behavioral deficits in *Chd8*^*N2373K/N2373K*^ mice. **a** Schema of behavioral experiments for male and female *Chd8*^*+/N2373K*^ and *Chd8*^*N2373K/N2373K*^ mice (3–4.5 months). **b** Locomotor activity in the Laboras test (familiar environment) as shown by distance moved by male and female *Chd8*^*+/N2373K*^ and *Chd8*^*N2373K/N2373K*^ mice. Full details of the statistical analyses are provided in Supplementary Table 1. (*n* = 16 mice [M/male-WT/wild-type], 13 [M-HT/heterozygote; 17 [M-HM/homozygote], 14 [F/female-WT], 12 [F-HT], 13 [F-HM], two-way ANOVA). **c** Locomotor activity in the open-field test (novel environment) as shown by distance moved. (*n* = 17 [M-WT], 13 [M-HT], 13 [M-HM], 17 [F-WT], 12 [F-HT], 16 [F-HM]), two-way ANOVA, one-way ANOVA within sex [indicated by blue asterisks]). **d** Time spent in center during the open-field test, as a measure of anxiety-like behavior. (*n* = 17 [M-WT], 13 [M-HT], 13 [M-HM], 17 [F-WT], 12 [F-HT], 16 [F-HM]), two-way ANOVA, one-way ANOVA within sex). **e** Light-dark test for anxiety-like behavior. (*n* = 18 [M-WT], 13 [M-HT], 14 [M-HM], 17 [F-WT], 13 [F-HT], 16 [F-HM], two-way ANOVA, one-way ANOVA within sex). **f** Elevated plus-maze test for anxiety-like behavior. (*n* = 18 [M-WT], 13 [M-HT], 14 [M-HM], 17 [F-WT], 13 [F-HT], 17 [F-HM], two-way ANOVA). **g** Self-grooming in Laboras cages. (*n* = 16 [M-WT], 13 [M-HT], 17 [M-HM], 14 [F-WT], 12 [F-HT], 13 [F-HM], two-way ANOVA, one-way ANOVA within sex). **h** Self-grooming in home cages. (*n* = 18 [M-WT], 11 [M-HT]; 14 [M-HM], 18 [F-WT], 13 [F-HT], 16 [F-HM], two-way ANOVA). **i** Courtship ultrasonic vocalizations (USVs). (*n* = 17 [M-WT], 13 [M-HT], 14 [M-HM], one-way ANOVA). **j** Direct social-interaction test. (*n* = 18 [M-WT], 12 [M-HT], 14 [M-HM], 18 [F-WT], 13 [F-HT], 17 [F-HM], two-way ANOVA, one-way ANOVA within sex). **k** Three-chamber social-interaction tests. S1, social target; O, object; S2, novel social target. Preference index: S1–O/S1 + O or S2–S1/S2 + S1. (*n* = 17 [M-WT], 13 [M-HT], 12 [M-HM], 17 [F-WT], 13 [F-HT], 16 [F-HM], two-way ANOVA, Student’s t-test [S1 vs. O or S1 vs. S2], one-way ANOVA within sex). **l** Summary of the behavioral results from *Chd8*^*+/N2373K*^ and *Chd8*^*N2373K/N2373K*^ males and females. Red/blue colors indicate fold changes (increases/decreases) in HT/HM mice relative to WT mice, and stars indicate significant differences between HT/HM and WT mice (one-way ANOVA followed by Dunnett’s test). Significance on two-way ANOVA is indicated as * (<0.05), ** (<0.01), *** (<0.001), or ns (not significant).

We next subjected *Chd8*^*+/N2373K*^ and *Chd8*^*N2373K/N2373K*^ males and females to a series of ASD-related behavioral tests assessing locomotor activity, anxiety-like behavior, repetitive behavior, and social interaction (Fig. 2a).

*Chd8*^*+/N2373K*^ mice on the hybrid background did not exhibit significant behavioral changes (Fig. 2b–k; summarized in Fig. 2l; Supplementary Table 1). This contrasts with the male-preponderant deficits observed in *Chd8*^*+/N2373K*^ mice on a pure background, suggesting that the hybrid background may buffer against the development of ASD-like behaviors.

In homozygous *Chd8*^*N2373K/N2373K*^ mice, females exhibited strong behavioral deficits, including open-field hypoactivity, anxiety-like behavior (open-field and light–dark but not elevated plus-maze tests), increased self-grooming in the Laboras cages (familiar environment) but not in the home cages (novel environment), and abnormally enhanced social interaction in the direct-interaction but not the three-chamber test, as compared with WT females (Fig. 2a–l). *Chd8*^*N2373K/N2373K*^ males showed qualitatively similar changes, but, apart from Laboras self-grooming, most measures did not reach statistical significance. Notably, Laboras self-grooming was significantly increased in mutant males relative to WT males, paralleling the robust increase observed in mutant females. Reintroducing outliers initially excluded by Grubbs’ test preserved the overall male–female difference patterns, though some female traits, namely open-field hypoactivity, open-field anxiety, and Laboras self-grooming, lost statistical significance (Supplementary Fig. 2; Supplementary Table 1).

These results indicate that a homozygous Chd8 mutation elicits stronger behavioral changes than a heterozygous mutation, establishing a robust causal link between CHD8 mutation and mouse behavioral deficits, a relationship that was less evident for core ASD-like behaviors (i.e., self-grooming) in earlier studies. Consistent with our effect-size analysis (Cohen’s d; Supplementary Table 1), *Chd8*^*N2373K/N2373K*^ females display more large effects (|d| ≥ 0.8) than males across the behavioral battery: in males, a large effect is mainly evident for Laboras grooming in the WT versus HM comparison, whereas in females large effects occur for Laboras grooming, open-field locomotor activity, open-field center time, and direct interaction (WT vs HM), as well as for direct interaction (WT vs HT). Together with the group variances (Supplementary Table 1), these findings suggest that increased mutation strength on the hybrid background attenuates the previously reported male behavioral bias and unmasks genuinely strong female phenotypes once a higher mutational threshold is exceeded.

### Brain volumes in Chd8^+/N2373K^ and Chd8^N2373K/N2373K^ mice

Mouse behavioral changes can be driven by multiple factors that shape brain development and function. To investigate these influences, we evaluated both male and female *Chd8*^*+/N2373K*^ and *Chd8*^*N2373K/N2373K*^ mice using assays that measured brain volume, cerebral blood flow, neuronal firing, synaptic transmission, and transcriptomic changes. CHD8 is known to regulate brain development and size, as evidenced by the macrocephaly commonly observed in autistic individuals with CHD8 mutations [1, 3, 4, 49, 52], although reduced expression of *Chd8* in mice gave mixed results in brain-size changes in gene dosage-sensitive manner [11, 13, 16, 17, 19, 20, 29, 31]. Additionally, the CHD8-N2373K mutation in the current study has been specifically linked to macrocephaly in both humans [4, 49] and mice [19].

**Fig. 3:**
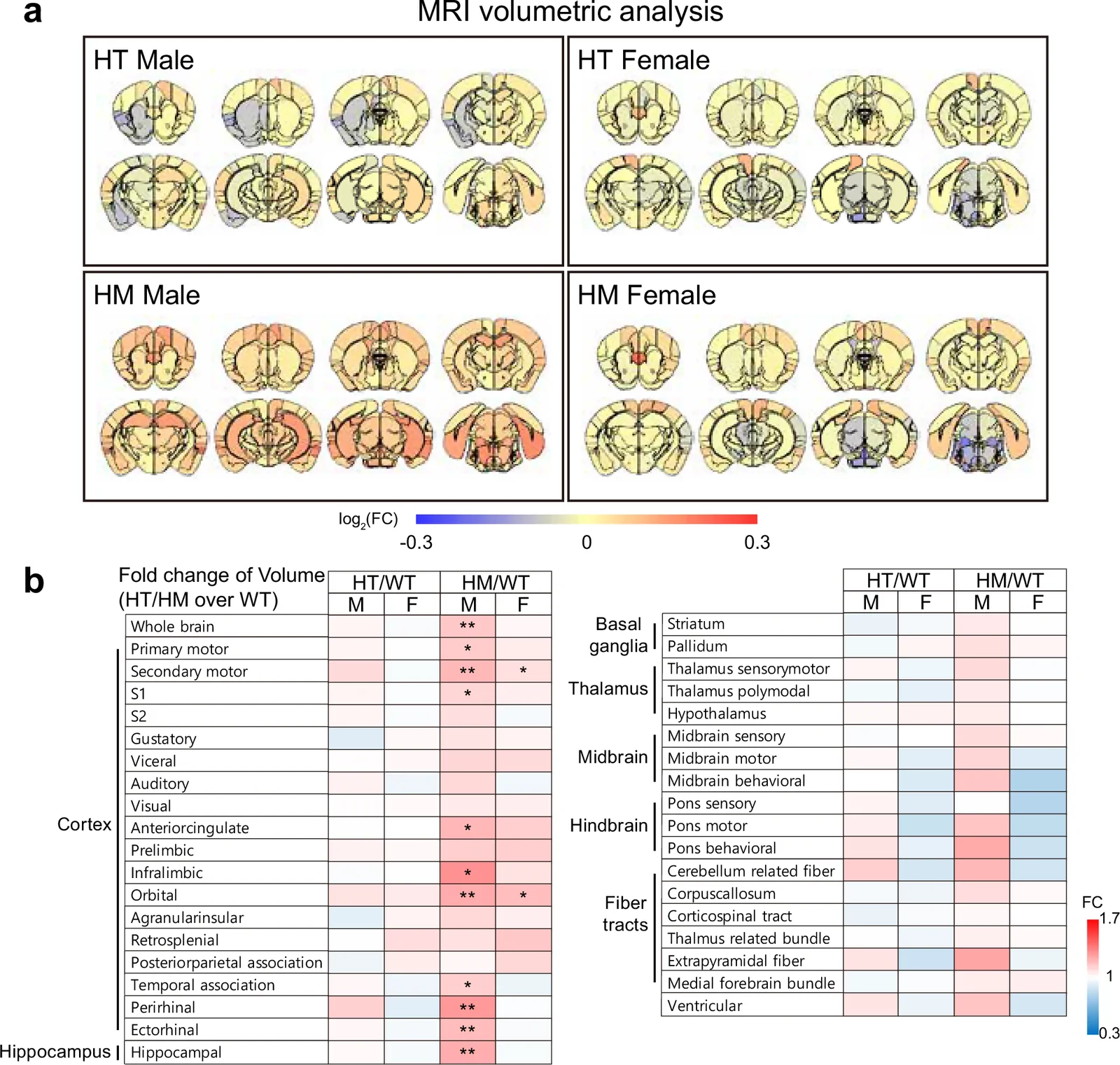
Brain volumes in *Chd8*^*+/N2373K*^ and *Chd8*^*N2373K/N2373K*^ males and females. **a** MRI analysis of alterations in anatomical brain volumes in *Chd8*^*+/N2373K*^ and *Chd8*^*N2373K/N2373K*^ male and female mice (~6–8 months). Red/blue colors indicate fold changes (increases/decreases) in HT/HM relative to WT mice. (*n* = 5 mice [M-WT], 5 [M-HT; 5 [M-HM], 5 [F-WT], 5 [F-HT], 5 [F-HM]). **b** Color-coded display of the altered anatomical brain volumes in the different brain regions shown in (a). Stars indicate significant differences between HT/HM mice and WT mice within males/females (one-way ANOVA with Dunnett’s test). The whole brain value represents the total brain volume calculated as the sum of all the indicated brain region volumes. Significance is indicated as * (<0.05), ** (<0.01), *** (<0.001), or ns (not significant).

We next used magnetic resonance imaging (MRI) to assess brain volumes in *Chd8*^*+/N2373K*^ and *Chd8*^*N2373K/N2373K*^ mice. In *Chd8*^*+/N2373K*^ mice, both males and females exhibited mixed trends in brain volume changes that did not reach significance (Fig. 3a, b), mirroring the lack of significant behavioral alterations (Fig. 2). In contrast, homozygous *Chd8*^*N2373K/N2373K*^ mice showed significant increases in brain volume across several regions (Fig. 3a, b). These volumetric increases were particularly prominent in mutant males, especially within cortical and hippocampal areas. Interestingly, while *Chd8*^*N2373K/N2373K*^ females also demonstrated increased volume in cortical regions, they showed decreasing tendencies in caudal regions, including the midbrain and hindbrain.

These results demonstrate that a homozygous Chd8 mutation intensifies macrocephaly, thereby more clearly linking Chd8 deficiency with increased brain volume. Specifically, both *Chd8*^*N2373K/N2373K*^ males and females exhibit similar increases in the cortical and hippocampal areas. However, they display divergent changes in the brain stem and hindbrain regions, with males showing increases and females showing decreases. It remains unclear whether these findings represent a gene dosage-dependent suppression of sexual dimorphism, particularly since *Chd8*^*+/N2373K*^ mice did not exhibit significant alterations, even though the overall patterns of sexual dimorphism across gene dosages tended to be similar.

### Cerebral blood volumes and flows in *Chd8*^*+/N2373K*^ and *Chd8*^*N2373K/N2373K*^ mice

To determine whether altered brain volumes are associated with functional changes, we measured cerebral blood volume (CBV) and cerebral blood flow (CBF) using functional magnetic resonance imaging (fMRI) with a whole BOLD–dynamic susceptibility contrast (BOLD-DSC) protocol that employs brief hypoxia stimuli to elicit robust BOLD responses and provides estimates of baseline CBV and CBF as proxies for resting perfusion and metabolic demand [53].

**Fig. 4:**
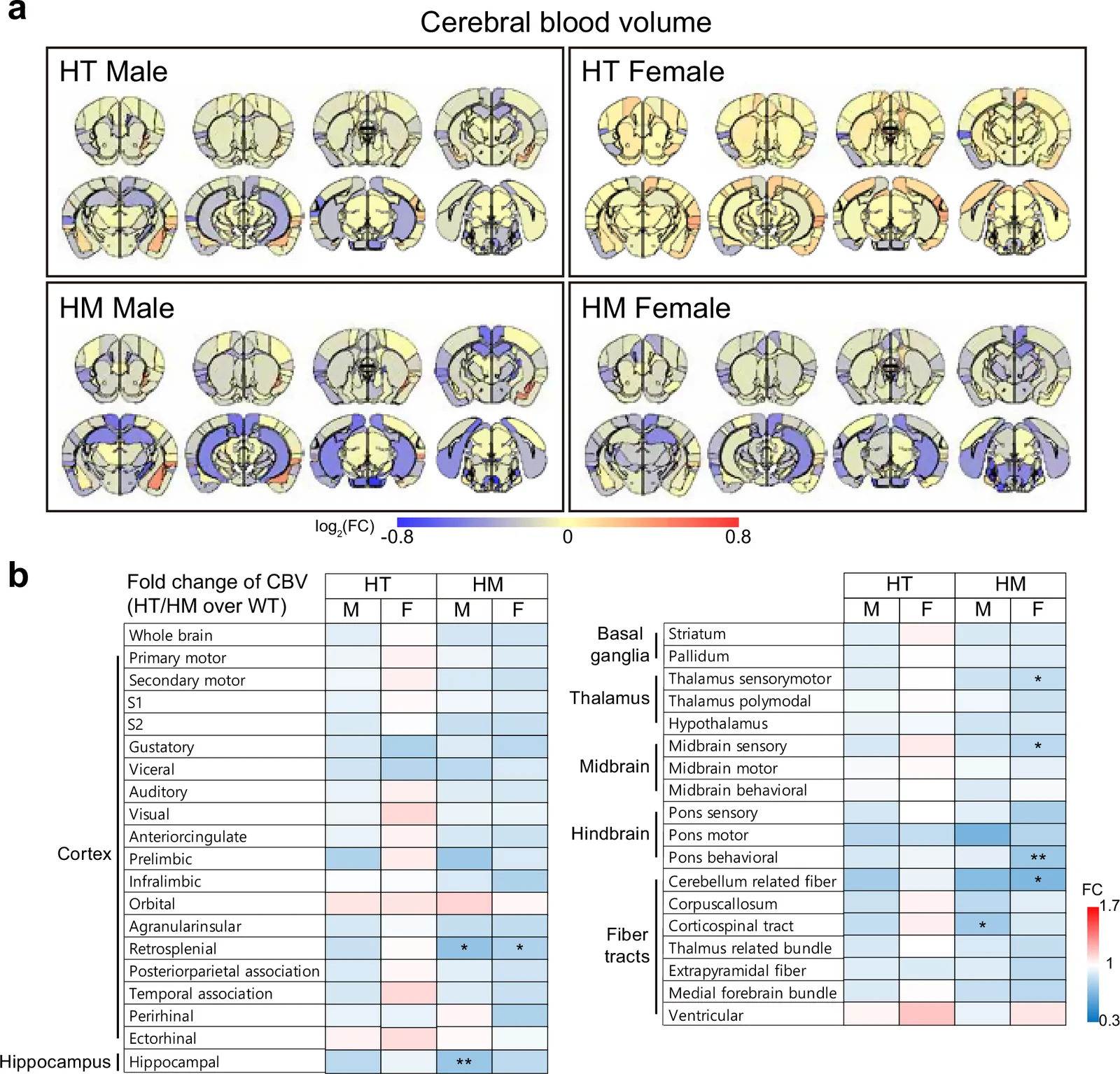
Cerebral blood volumes in *Chd8*^*+/N2373K*^ and *Chd8*^*N2373K/N2373K*^ males and females. **a** Blood volumes in different brain regions of *Chd8*^*+/N2373K*^ and *Chd8*^*N2373K/N2373K*^ male and female mice (~4–5 months), as measured by fMRI responding to 5-sec nitrogen stimulus under anesthesia (isoflurane). Red/blue colors indicate fold changes (increases/decreases) in HT/HM relative to WT mice. (*n* = 5 mice [M-WT], 5 [M-HT; 5 [M-HM], 5 [F-WT], 5 [F-HT], 5 [F-HM]). **b** Color-coded display of the altered cerebral blood volumes in the different brain regions shown in (a). Stars indicate significant differences between HT/HM mice and WT mice within males/females (one-way ANOVA with Dunnett’s test). The whole brain value represents the average blood volume across all the indicated brain regions. Significance is indicated as * (<0.05), ** (<0.01), *** (<0.001), or ns (not significant).

In *Chd8*^*+/N2373K*^ mice, males exhibited slight, non-significant decreases in CBV across several brain regions (Fig. 4a, b), while females showed mixed patterns of small increases and decreases, differing from the primarily decreasing trend seen in males. In contrast, both male and female *Chd8*^*N2373K/N2373K*^ mice displayed similar decreases in CBV in largely non-overlapping regions of male and female mutants, except for the retrosplenial cortex (Fig. 4a, b). Cerebral blood flow analyses produced similar patterns (Supplementary Fig. 3). As illustrated by the hypoxia-evoked CBV time courses in the hippocampus (Supplementary Fig. 4a), group differences were largely reflected in the magnitude of the peak response, whereas the overall temporal profile of the vascular response was preserved across genotypes and sexes, arguing against major alterations in vascular reactivity.

These results collectively suggest that a homozygous Chd8 mutation leads to a more pronounced suppression of CBV and CBF. These CBV/CBF differences are unlikely to arise from systematic differences in systemic physiology or anesthetic depth, as respiratory rates—an indicator of anesthetic depth—were comparable across groups during imaging. Due to the insignificant CBF differences observed in Chd8^+/N2373K^ mice, it remains unclear whether heightened mutational strength influences sexually dimorphic blood flow patterns. However, the contrasting trends in blood flow between Chd8^+/N2373K^ males and females converge into a shared, significant decrease in both sexes in the homozygous mutants.

To investigate the relationship between regional CBV and MRI-derived brain volume (Fig. 4 vs. Figure 3), we performed correlation and ANCOVA analyses (Supplementary Fig. 4b–e). No significant associations were detected after correction for multiple comparisons, although nominal negative correlations were observed only in prelimbic and retrosplenial cortices. Lastly, ANOVA versus ANCOVA comparisons including total brain volume and body weight as covariates (Supplementary Fig. 4e) yielded broadly similar patterns of group p values, indicating that regional CBV reductions are unlikely to be explained by variation in local regional volume, overall brain size, or body weight.

### Neuronal firing and brain rhythms in *Chd8*^*+/N2373K*^ and *Chd8*^*N2373K/N2373K*^ mice

**Fig. 5:**
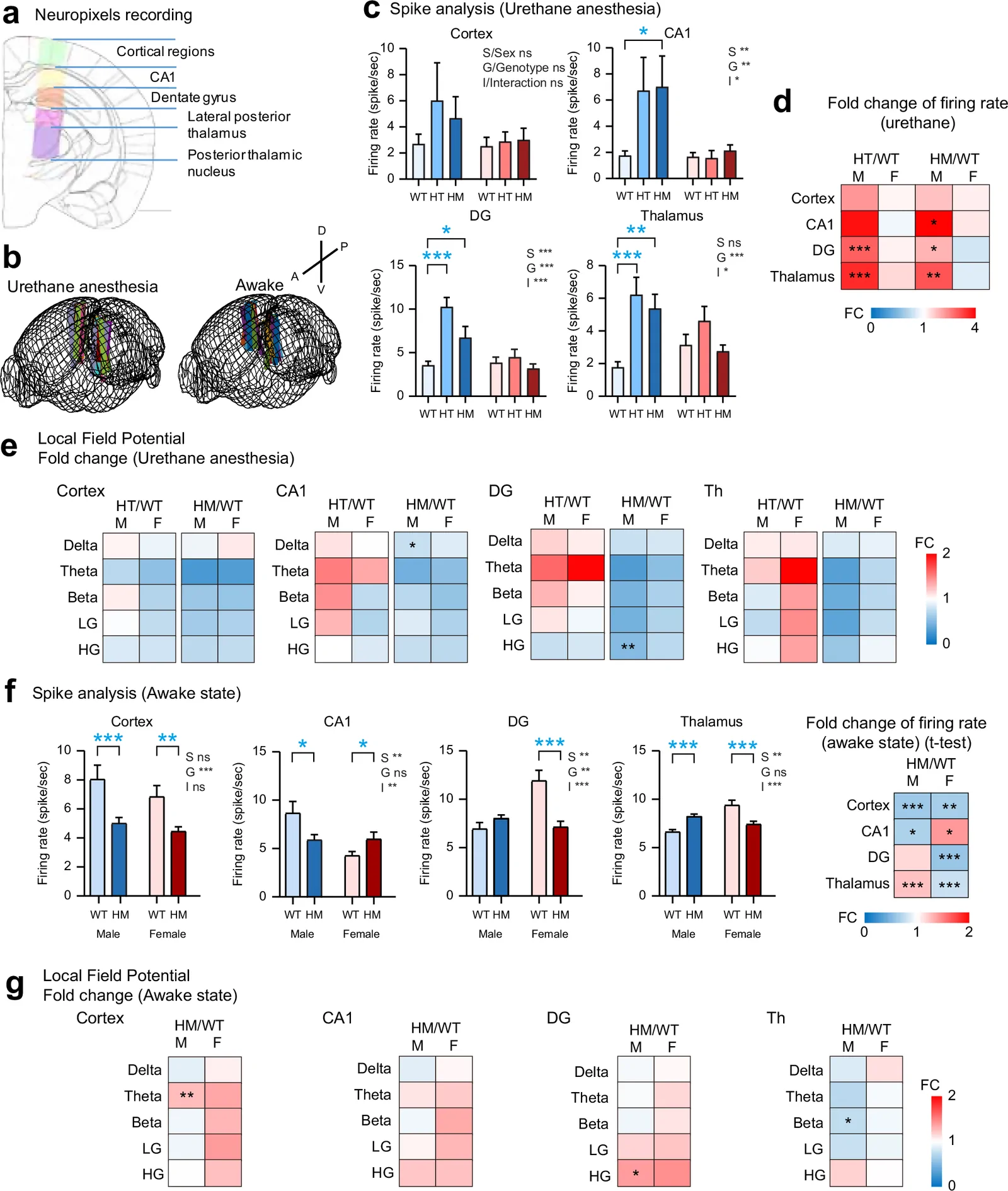
Neuronal firing and brain rhythms in *Chd8*^*+/N2373K*^ and *Chd8*^*N2373K/N2373K*^ males and females. **a** Schema of Neuropixels recording of neuronal firing in multiple brain regions of *Chd8*^*+/N2373K*^ and *Chd8*^*N2373K/N2373K*^ males and females (9–16 weeks), including the cortex (retrosplenial and visual), hippocampus (CA1 and dentate gyrus/DG), and thalamus (lateral posterior and posterior thalamic). Scale bar, 1 mm. **b** Post-hoc histological 3D reconstruction of Neuropixels probe tracks, with different colors representing individual animals. Tracks are shown for the urethane anesthesia experiment (left) and the head-fixed awake experiment (right). **c** Neuronal firing rates in multiple brain regions (cortex, hippocampal CA1 and DG, and thalamus), measured by Neuropixels recordings under urethane anesthesia. (Cortex, *n* = 28 neurons from 9 mice [M-WT], 22, 8 [M-HT], 15, 9 [M-HM], 27, 5 [F-WT], 26, 5 [F-HT], 20, 5 [F-HM]; CA1, 40, 9 [M-WT], 21, 8 [M-HT], 24, 9 [M-HM], 48, 5 [F-WT], 23, 5 [F-HT], 46, 5 [F-HM]; DG 185, 9 [M-WT], 125, 8 [M-HT], 68, 9 [M-HM], 76, 5 [F-WT], 66, 5 [F-HT], 122, 5 [F-HM]; Th, 100, 9 [M-WT], 116, 8 [M-HT], 136, 9 [M-HM], 121, 5 [F-WT], 98, 5 [F-HT], 205, 5 [F-HM], two-way ANOVA, one-way ANOVA [within-sex comparison of WT, HT, and HM]). **d** Ratios of neuronal firing rates (HT/WT and HM/WT) in multiple brain regions (cortex, CA1, DG, and thalamus; urethane). Red/blue colors indicate fold changes (increases/decreases) in HT/HM mice relative to WT mice, and stars indicate significant differences between HT/HM and WT mice within males/females. (*n* = 9 [M-WT],8 [M-HT], 9 [M-HM], 5 [F-WT], 5 [F-HT], and 9 [F-HM], one-way ANOVA with Dunnett’s test). **e** Ratios of local rhythms (HT/WT and HM/WT) in multiple brain regions (cortex, CA1, DG, and cortex) of *Chd8*^*+/N2373K*^ and *Chd8*^*N2373K/N2373K*^ males and females (9–16 weeks; urethane). Red/blue colors indicate fold changes (increases/decreases) in HT/HM mice relative to WT mice, and stars indicate significant differences between HT/HM and WT mice (one-way ANOVA with Dunnett’s test). (*n* = 9 [M-WT], 8 [M-HT], 8 [M-HM], 5 [F-WT], 6 [F-HT], and 8 [F-HM], one-way ANOVA with Dunnett’s test). **f** Awake-state neuronal firing rates in multiple brain regions (cortex, CA1, DG, and thalamus) and ratios of neuronal firing rates (HM/WT). (Firing rates: Cortex, 106 neurons from 3 mice [M-WT], 212, 3 [M-HM], 154, 4 [F-WT], 257, 3 [F-HM]; CA1, 62, 3 [M-WT], 131, 3 [M-HM], 211, 4 [F-WT], 116, 3 [F-HM]; DG, 108, 3 [M-WT], 202, 3 [M-HM], 153, 4 [F-WT], 159, 3 [F-HM]; Th, 671, 3 [M-WT], 777, 3 [M-HM], 549, 4 [F-WT], 760, 3 [F-HM], one-way ANOVA with Dunnett’s test; Ratios of firing rates: *n* = 6 hemispheres from 3 mice [M-WT], 6, 3 [M-HM], 6, 3 [F-WT], and 6, 3 [F-HM], two-way ANOVA, unpaired t-test [within-sex comparison of WT and HM]). **g** Awake-state ratios of local rhythms (HM/WT) in multiple brain regions (cortex, CA1, DG, and cortex) of *Chd8*^*+/N2373K*^ and *Chd8*^*N2373K/N2373K*^ males and females (9–16 weeks). (Cortex, *n* = 4 hemispheres from 3 mice [M-WT], 3, 3 [M-HM], 5, 4 [F-WT], 4, 3 [F-HM]; CA1, 4, 3 [M-WT], 3, 3 [M-HM], 7, 4 [F-WT], 4, 3 [F-HM]; DG, 4, 3 [M-WT], 3, 3 [M-HM], 6, 4 [F-WT], 4, 3 [F-HM]; Th, 4, 3 [M-WT], 3, 3 [M-HM], 6, 4 [F-WT], 4, 3 [F-HM], two-way ANOVA, unpaired t-test [within-sex comparison of WT and HM]). Significance is indicated as * (<0.05), ** (<0.01), *** (<0.001), or ns (not significant).

We next measured neuronal firing and local brain rhythms in various brain regions of male and female *Chd8*^*+/N2373K*^ and *Chd8*^*N2373K/N2373K*^ mice using Neuropixels probes under urethane anesthesia, which can stably measure high-density neuronal activity simultaneously in multiple brain regions of live animals [54]. Specifically, we measured neuronal activity in the cortex (retrosplenial or primary visual cortex, depending on the exact probe trajectory), hippocampus (CA1 and dentate gyrus/DG), and thalamus (lateral posterior and posterior thalamic regions) (Fig. 5a, b).

In *Chd8*^*+/N2373K*^ males under urethane, neuronal firing frequency increased by approximately 2–3-fold in the hippocampal dentate gyrus and thalamus, while *Chd8*^*+/N2373K*^ females did not show significant changes (Fig. 5c, d). Similarly, *Chd8*^*N2373K/N2373K*^ males exhibited increased neuronal firing in both the hippocampus (CA1 and DG) and thalamus, whereas homozygous mutant females remained unchanged. These results indicate that increased neuronal firing is a male-specific effect and is not affected by a stronger CHD8 mutation.

We next analyzed local field potentials (LFPs) to assess local brain rhythms (Supplementary Fig. 5a-d). In *Chd8*^*+/N2373K*^ males under urethane, LFP power showed increasing trends across multiple frequency bands (delta, theta, beta, low-gamma, and high-gamma) in the cortex, hippocampus (CA1/DG), and thalamus, whereas *Chd8*^*+/N2373K*^ females exhibited mixed trends (Fig. 5e). However, none of these changes reached statistical significance. In contrast, *Chd8*^*N2373K/N2373K*^ mice displayed decreasing trends in LFP power in both sexes with some male regions (e.g., DG) reaching significance, differing from the patterns seen in heterozygous mice (Fig. 5e).

In a separate set of head-fixed awake recordings targeting the same cortical, hippocampal, and thalamic regions from *Chd8*^*N2373K/N2373K*^ mice, male-specific increases in neuronal firing was observed in a subset of the brain regions (DG and thalamus but not CA1) (Fig. 5f), being partly similar to the results from anesthetized *Chd8*^*N2373K/N2373K*^ mice. Notably, the cortex displayed similar decreases in neuronal firing in male and female mutants, while the CA1 region showed a female (not male)-specific increase. These results indicate brain region-differential effects of urethane on neuronal firing. In addition, the switch in directionality from increased (anesthetized) to decreased (awake) cortical firing, and the emergence of a female-specific CA1 increase only in awake mice, likely reflects the well-known modulatory effects of urethane on excitatory and inhibitory neurotransmission [55, 56], which can differentially mask or unmask region- and circuit-specific firing alterations depending on the local balance of excitation and inhibition [57–59]. Indeed, cortical responses in awake mice are dominated by inhibition and are more temporally restricted than under urethane anesthesia, where prolonged excitatory drive predominates [60], consistent with a state-dependent shift in excitation–inhibition balance underlying the observed directionality switch.

When LFPs were analyzed in awake mice, LFP powers were tended to decrease in the thalamus of both sexes and in male (but not female) cortex, CA1, and DG (Fig. 5g; Supplementary Fig. 6), being qualitatively similar to the results from anesthetized mice, although these results were largely statistically insignificant. Overall, these findings indicate that the homozygous *Chd8*^*N2373K/N2373K*^ mutation does not further elevate neuronal firing as compared with the heterozygous *Chd8*^*+/N2373K*^ mutation under anesthesia, being already pronounced in heterozygous males especially in the DG and thalamus, as further supported by the awake-mouse results. Whether gene dosage suppresses sexual dimorphism in LFP remains unclear due to the largely insignificant LFP changes in heterozygous and homozygous mice under anesthesia, although a trend toward suppression is apparent especially in the thalamus.

### Synaptic transmission in *Chd8*^*+/N2373K*^ and *Chd8*^*N2373K/N2373K*^ mice

We next assessed excitatory and inhibitory synaptic transmission in hippocampal CA1 pyramidal neurons from *Chd8*^*+/N2373K*^ and *Chd8*^*N2373K/N2373K*^ mice, given that prior work in a pure genetic background reported female-specific increases in inhibitory transmission in *Chd8*^*+/N2373K*^ mice [19].

On the hybrid background used here, *Chd8*^*+/N2373K*^ males showed no detectable changes in miniature excitatory postsynaptic currents (mEPSCs), while *Chd8*^*+/N2373K*^ females showed a non-significant tendency toward reduced mEPSC frequency (Supplementary Fig. 7a). In addition, miniature inhibitory postsynaptic currents (mIPSCs) remained unchanged in both sexes of *Chd8*^*+/N2373K*^ mice (Supplementary Fig. 7b), differing from earlier findings in a pure background [19], which is likely to be caused by a change in the genetic background.

In homozygous *Chd8*^*N2373K/N2373K*^ mice, mEPSCs and mIPSCs were unaltered in both sexes (Supplementary Fig. 7a, b). Moreover, spontaneous excitatory and inhibitory currents (sEPSCs and sIPSCs) were unaltered in all groups (Supplementary Fig. 7c, d).

In additional recordings from layer 2/3 and layer 5 pyramidal neurons in the medial prefrontal cortex (mPFC) and from dorsal striatal neurons, sEPSC and sIPSC properties were generally normal in Chd8N2373K/N2373K mice, with the exception of an increase in sIPSC frequency in layer 5 pyramidal neurons (Supplementary Fig. 8a–f).

Collectively, these results indicate that the heterozygous *Chd8* mutation does not affect synaptic properties in the hippocampus of mice on the hybrid background, underscoring, together with previous results from a pure background with female-specific inhibitory synaptic increases [19], that genetic background strongly shapes the expression of synaptic changes. The present data further demonstrate that the homozygous Chd8 mutation does not alter baseline excitatory or inhibitory synaptic transmission in the hippocampus, mPFC, or striatum. As such, these findings do not address whether increased Chd8 mutational strength intensifies synaptic phenotypes or contributes to synaptic sexual dimorphism.

### Transcriptomic changes in *Chd8*^*+/N2373K*^ and *Chd8*^*N2373K/N2373K*^ mice

**Fig. 6:**
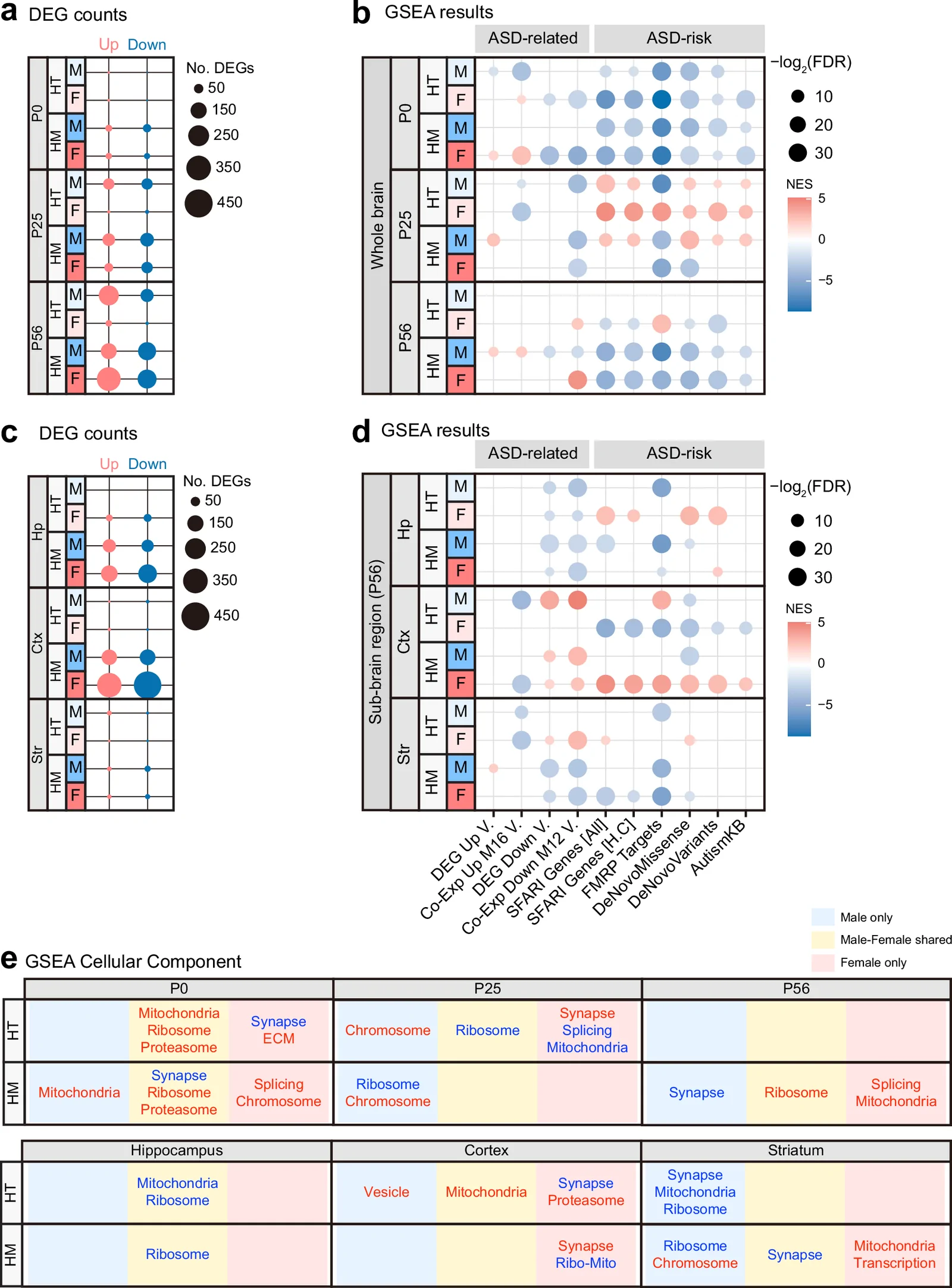
Transcriptomic changes in *Chd8*^*+/N2373K*^ and *Chd8*^*N2373K/N2373K*^ males and females. **a**, **b** Differentially expressed gene (DEG; a) and Gene set enrichment analysis (GSEA; b) analyses of transcriptomes from whole brains of *Chd8*^*+/N2373K*^ and *Chd8*^*N2373K/N2373K*^ males and females at P0, P25, and P56. The GSEA used ASD-related sets (DEG Up Voineagu, Co-Exp Up M16 Voineagu, DEG Down Voineagu, Co-Exp Down M12 Voineagu) and ASD-risk gene sets (SFARI Genes [All, High-confidence], FMRP Targets, DeNovoMissense, DeNovoVariants, and AutismKB). Up/Down, up/down regulations; P0/25/56, postnatal day 0/25/56; FDR, false discovery rate; NES, normalized enrichment score. (*n* = 5 mice [WT-male-P0], 5 [WT-female-P0], 5 [HT-male-P0], 5 [HT-female-P0], 5 [HM-male-P0], 5 [HM-female-P0]), 5 [WT-male-P25], 5 [WT-female-P25], 5 [HT-male-P25], 5 [HT-female-P25], 5 [HM-male-P25], 5 [HM-female-P25]), 5 [WT-male-P56], 5 [WT-female-P56], 5 [HT-male-P56], 5 [HT-female-P56], 5 [HM-male-P56], 5 [HM-female-P56], FDR < 0.05). **c**, **d** DEG and GSEA analyses of transcriptomes from three different brain regions (hippocampus/Hp, cortex/Ctx, and striatum/Str) of *Chd8*^*+/N2373K*^ and *Chd8*^*N2373K/N2373K*^ males and females at P56. (*n* = 5 [WT-male-Hp], 5 [WT-female-Hp], 5 [HT-male-Hp], 5 [HT-female-Hp], 5 [HM-male-Hp], 5 [HM-female-Hp]), 5 [WT-male-Ctx], 5 [WT-female-Ctx], 5 [HT-male-Ctx], 5 [HT-female-Ctx], 5 [HM-male-Ctx], 5 [HM-female-Ctx]), 5 [WT-male-Str], 5 [WT-female-Str], 5 [HT-male-Str], 5 [HT-female-Str], 5 [HM-male-Str], 5 [HM-female-Str], FDR < 0.05). **e** A summary of the biological functions associated with the transcriptomic changes observed in *Chd8*^*+/N2373K*^ and *Chd8*^*N2373K/N2373K*^ mice across both sexes. These functions highlight the effects induced by increased gene deletion dosage and reveal both shared and distinct changes between males and females (see Supplementary Figs. 8–11 for details). For simplicity, only the GSEA results in the Gene Ontology—Cellular Component (GO-CC) domain are summarized. Male-only, female-only, and male/female-shared changes are indicated in light blue, pink, and yellow backgrounds, respectively, while up- and downregulated gene sets are marked in red and blue.

To explore molecular mechanisms associated with the phenotypic changes in *Chd8*^*+/N2373K*^ and *Chd8*^*N2373K/N2373K*^ males and females, we next performed transcriptomic analyses at three postnatal stages (P0, P25, and P56) using whole-brain samples and in adults across three regions (hippocampus, cortex, and striatum) (Fig. 6; Supplementary Table 2). Our RNA-Seq analyses mainly involved two approaches; analyses of differentially expressed genes (DEGs) and gene set enrichment analysis (GSEA) [61]. The latter used ASD-related gene sets (DEG Up Voineagu, Co-Exp Up M16 Voineagu, DEG Down Voineagu, Co-Exp Down M12 Voineagu) [62, 63], ASD-risk gene sets (SFARI Genes [All, High-confidence] [64], FMRP Targets [63, 65], DeNovoMissense [63, 66], DeNovoVariants [63, 66], and AutismKB [67, 68]), and biological function-related gene sets in the Molecular Signature Database (MSigDB).

At P0, only small numbers of DEGs were observed in *Chd8*^*+/N2373K*^ mice without apparent difference in males and females (Fig. 6a; Supplementary Table 3 [all DEG results]). In *Chd8*^*N2373K/N2373K*^ mice, similar results were also observed, although DEG numbers were slightly increased. This suggests that gene deletion dosage increases DEG numbers but does not affect sexual dimorphism.

In the GSEA for P0, *Chd8*^*+/N2373K*^ and *Chd8*^*N2373K/N2373K*^ males and females showed largely similar changes in ASD-related/risk gene sets, with the *Chd8*^*N2373K/N2373K*^ mice showing slightly stronger ASD-like patterns; i.e., *Chd8*^*N2373K/N2373K*^ males and females showing stronger ASD-like patterns such as stronger upregulation of ASD-related genes (DEG Up and Co-Exp Up) and stronger downregulation of ASD-related genes (DEG Down, Co-Exp Down) and ASD-risk genes (i.e., SFARI) (Fig. 6b; Supplementary Table 4 [all GSEA results]).

At P25, *Chd8*^*+/N2373K*^ males showed stronger changes in DEGs (both up and down) as compared with *Chd8*^*+/N2373K*^ females (Fig. 6a). Intriguingly, in *Chd8*^*N2373K/N2373K*^ mice, DEG numbers being similar in males and females, suggestive of gene dosage-dependent suppression of sexual dimorphism in DEG number. Accordingly, the GSEA results from *Chd8*^*+/N2373K*^ mice showed stronger ‘reverse-ASD’ patterns (changes opposite to those observed in ASD) in females relative to males; i.e., downregulation of Co-Exp UP genes and upregulation of most ASD-risk gene sets (SFARI and FMRP Targets) (Fig. 6b). However, this strong reverse-ASD pattern in *Chd8*^*+/N2373K*^ females was changed to ASD-like patterns in *Chd8*^*N2373K/N2373K*^ females, partly similar to the male pattern, again suggesting gene dosage-dependent suppression of sexual dimorphism.

At P56, similar gene dosage-dependent suppression of sexual dimorphism was observed in both DEG numbers and GSEA results from *Chd8*^*+/N2373K*^ and *Chd8*^*N2373K/N2373K*^ mice (Fig. 6a, b), indicating that gene dosage-dependent suppression of sexual dimorphism is detectable at ~P25 and persists into adulthood.

In DEG analyses for different brain regions (hippocampus, cortex, and striatum) at P56, brain region-specific changes were observed. The hippocampus and cortex, but not the striatum, showed DEG patterns that are similarly robust in *Chd8*^*N2373K/N2373K*^ males and females (Fig. 6c), mirroring patterns observed in the whole brain. GSEA analyses for ASD-related/risk gene sets revealed that the hippocampus and striatum, but not the cortex, showed stronger gene dosage-dependent suppression of sexual dimorphism (Fig. 6d), partially aligning with results from both whole-brain analyses and DEG findings.

Collectively, these findings indicate that a homozygous Chd8 mutation amplifies transcriptomic alterations, as evidenced by a higher number of DEGs and more pronounced ASD-like GSEA changes, particularly at postnatal day 56 and in the striatum. Moreover, gene dosage–dependent suppression of sexual dimorphism emerges in a developmental stage– and brain region–specific manner, again most notably at P56 (in both DEG and GSEA analyses) and within the striatum (via GSEA). For example, the robust reverse-ASD pattern observed in *Chd8*^*+/N2373K*^ females across the whole brain shifts to an ASD-like pattern shared by both male and female *Chd8*^*N2373K/N2373K*^ mice, with a similar transition noted in the striatum at P56.

### Biological functions linked to transcriptomic changes in *Chd8*^*+/N2373K*^ and *Chd8*^*N2373K/N2373K*^ mice

To better understand the biological basis of the abovementioned transcriptomic changes (i.e., ASD-related/risk gene expressions and gene dosage-dependent suppression of sexual dimorphism), we performed GSEA using biological function-related gene sets in the gene ontology (GO) cellular component/CC and biological process/BP domains (summarized Fig. 6e; see Supplementary Figs. 9–12 for details**;** Supplementary Table 4).

At P0, robust ASD-like transcriptomic changes were observed in the whole brains of both *Chd8*^*+/N2373K*^ and *Chd8*^*N2373K/N2373K*^ males and females (Fig. 6b). Consistent upregulation of ribosomal and proteasomal genes was evident across all genotypes and sexes (Fig. 6e; Supplementary Figs. 9–12). Notably, while mitochondrial upregulation was present in *Chd8*^*+/N2373K*^ mice, this shifted to synaptic downregulation in *Chd8*^*N2373K/N2373K*^ mice. In addition, distinct male-only and female-only gene regulations were identified. These findings indicate that at P0, gene regulation is influenced by both genotype and sex, exhibiting both shared and unique patterns.

At P25, *Chd8*^*+/N2373K*^ females exhibit pronounced reverse-ASD patterns across the whole brain (Fig. 6b). Notably, these females display unique changes; synaptic upregulation paired with mitochondrial and splicing downregulation (Fig. 6e; Supplementary Figs. 9–12). These alterations may contribute to female neuroprotection, aligning with previous reports of robust, female-specific increases in inhibitory synaptic transmission in *Chd8*^*+/N2373K*^ females on a pure genetic background [19].

At P56, *Chd8*^*N2373K/N2373K*^ mice exhibit pronounced ASD-like transcriptomic patterns across the whole brain, whereas *Chd8*^*+/N2373K*^ mice of both sexes did not display these alterations (Fig. 6b). Alongside the observed age-dependent decline in ASD-related/risk gene expression from P0 to P25 and P56, this suggests a spontaneous normalization of ASD-related gene expression in *Chd8*^*+/N2373K*^ mice, which is consistent with their largely normal behavioral phenotypes. In contrast, biological changes remain prominent in *Chd8*^*N2373K/N2373K*^ mice, characterized by a shared upregulation of ribosomal genes and sex-specific alterations; namely, synaptic downregulation in males and splicing and mitochondrial downregulations in females (Fig. 6e; Supplementary Figs. 9–12).

In specific brain regions, particularly the hippocampus and striatum in *Chd8*^*N2373K/N2373K*^ males and females, which show shared ASD-like changes (Fig. 6d), shared biological alterations were observed in *Chd8*^*N2373K/N2373K*^ mice but not in *Chd8*^*+/N2373K*^ mice; downregulations of ribosomal and synaptic genes were evident in the hippocampus and striatum, respectively (Fig. 6e).

Notably, the downregulated mitochondrial and splicing gene sets unique to P25 *Chd8*^*+/N2373K*^ females (whole brain) were no longer detectable in P56 *Chd8*^*+/N2373K*^ females, but emerged in P56 *Chd8*^*N2373K/N2373K*^ females as upregulated gene sets (Fig. 6e). These opposite changes might underlie the contrasting female-protective and female-susceptible phenotypes/behaviors at P25 and P56 (e.g., reverse-ASD and ASD-like transcriptomes, respectively; Fig. 6b). To determine whether these contrasting changes involve a similar set of synaptic genes, we conducted leading edge analyses [61]. The results indicated that many shared splicing and mitochondrial genes (i.e., Snrpg [small nuclear ribonucleoprotein G] and Txnl4a [thioredoxin-like protein 4 A]; key components of the spliceosome) were involved in the opposite changes (Supplementary Fig. 13).

On a similar note, the synaptic upregulation in P25 *Chd8*^*+/N2373K*^ females (whole brain) contrasted with the strong synaptic downregulation shared in P56 *Chd8*^*N2373K/N2373K*^ males and females (striatum). Our results, however, indicated that distinct synaptic genes were involved in these cases; i.e., Grin2a, Grin2b, and Shisa6 (regulators of NMDARs and AMPARs) in P25 *Chd8*^*+/N2373K*^ females and Ntng2 and Lrfn2 (synaptic adhesion molecules) in P56 *Chd8*^*N2373K/N2373K*^ mice (Supplementary Fig. 14), likely because we compared two different brain regions (whole brain vs. striatum).

Collectively, these results indicate that a homozygous Chd8 mutation amplifies biological changes, most notably at P56 in the whole brain and striatum, as reflected by an increased number of gene-set clusters. Increased Chd8 mutation strength appears to suppress, and in specific gene sets partially reverse, sexual dimorphism in biological functions. For example, the synaptic upregulation along with the splicing and mitochondrial downregulations that are uniquely observed in P25 *Chd8*^*+/N2373K*^ females (but not in males) are either eliminated (synapse) or reversed (splicing and mitochondria) in P56 *Chd8*^*N2373K/N2373K*^ females.

## Discussion

By utilizing a hybrid genetic background to circumvent the lethality typically associated with homozygous Chd8 mutations, we generated both heterozygous (*Chd8*^*+/N2373K*^) and homozygous (*Chd8*^*N2373K/N2373K*^) mice, enabling a systematic comparison of gene dosage effects on phenotypic severity and male-female phenotypes. Our results indicate that increasing the severity of Chd8 mutations intensifies ASD-related phenotypes and attenuates male–female behavioral differences while reducing sexually dimorphic patterns across several neurobiological domains (i.e., cerebral blood flow, brain rhythms, and transcriptomes).

Behaviorally, *Chd8*^*+/N2373K*^ mice on a pure genetic background display predominantly male-biased deficits [19], whereas in the hybrid background, *Chd8*^*N2373K/N2373K*^ mice exhibit either male–female shared repetitive behaviors or even female-preponderant deficits. This shift likely reflects the combined influence of the altered genetic background and the increased mutation strength. Although direct behavioral comparisons in the hybrid background did not conclusively demonstrate that increased mutational strength suppresses sexual dimorphism, primarily because *Chd8*^*+/N2373K*^ mice showed minimal behavioral changes, other phenotypic assessments (including brain volume, cerebral blood flow, neuronal firing, synaptic transmission, and transcriptomic profiles) strongly indicate that a higher mutation strength attenuates sexually dimorphic traits. These findings underscore mutation strength as a critical factor in modulating sex differences.

A potential framework for interpreting these results is the female protective effects (FPE) hypothesis, which posits that females require a higher mutational threshold to manifest neurodevelopmental disorders such as ASD [69–75]. Although the FPE hypothesis offers intuitive and testable predictions, direct neurobiological evidence from animal models of ASD and other neurodevelopmental disorders has been limited. While many mouse models of ASD display sexually dimorphic phenotypes, the underlying mechanisms remain elusive [63, 75–84]. Our current results, together with the previous report [19], provides evidence that can be aligned with the FPE hypothesis: heterozygous mutants (pure genetic background) frequently exhibit pronounced sex differences, whereas homozygous mutants (hybrid genetic background) display more convergent phenotypes. This suggests that once the genetic insult surpasses a female-specific protective threshold, female phenotypes become comparable to, or even more severe than, those in males (i.e., equally robust self-grooming or even stronger other behavioral deficits in *Chd8*^*N2373K/N2373K*^ females). FPEs are thought to contribute to the marked sex ratios observed in various neurodevelopmental disorders, including ASD [69–74, 78–83], ADHD [85, 86], and schizophrenia [87, 88]. Moreover, CHD8 is implicated in ASD [1–7] and many other neurodevelopmental conditions, such as intellectual disability, developmental delay, ADHD, and schizophrenia [8, 9]. Thus, our findings may offer broader insights into the mechanisms underlying sexual dimorphism across multiple neurodevelopmental disorders.

Our analyses of brain anatomy and cerebral blood flow offer further insights into CHD8’s role in brain development and function. Homozygous *Chd8*^*N2373K/N2373K*^ mice exhibit increased brain volumes compared to heterozygous *Chd8*^*+/N2373K*^ mice, suggesting that a strong reduction in Chd8 expression drives macrocephaly, a finding consistent with previous studies in mice expressing approximately 35% of wild-type CHD8 levels [31]. Regarding sex differences, both mutant males and females show similar increases in the cortical and hippocampal regions. However, they diverge in the brain stem and hindbrain regions, with males displaying increases and females showing decreases. In contrast, cerebral blood flow is similarly reduced across all brain regions in both sexes of *Chd8*^*N2373K/N2373K*^ mice. This suggest that there is a disconnect between the anatomical macrocephaly induced by the Chd8 mutation and functional brain activity and that cerebral blood flow shows a stronger gene dose-dependent suppression of sexual dimorphism than brain volume.

Data on neuronal firing and synaptic transmission provide further insight into the functional impact of Chd8 mutations. Under urethane anesthesia, both heterozygous (*Chd8*^*+/N2373K*^) and homozygous (*Chd8*^*N2373K/N2373K*^) males show similarly elevated neuronal firing across multiple regions, indicating that stronger mutation does not further increase this abnormality, whereas females show minimal firing changes but display LFP power reductions that resemble those in males, consistent with attenuated sexual dimorphism in brain rhythms. In awake recordings, the male-specific increase in firing is again apparent in DG and thalamus of Chd8N2373K/N2373K mice, while both sexes show reduced firing in the cortex and a shared reduction in thalamic LFP power, suggesting a brain region–specific suppression of sexual dimorphism, although most LFP changes did not reach statistical significance in either anesthetized or awake conditions.

Our transcriptomic analyses further illuminate CHD8 function by extending previous findings on age- and brain region-specific differences in Chd8-mutant mice [13, 16, 20, 28, 40, 89]. Moreover, increased CHD8 mutation strength suppresses sexually dimorphic transcriptomic patterns, as revealed by both DEG and GSEA analyses. These effects vary by developmental stage and brain region, with the most pronounced changes at P56 and in the striatum. For example, the synaptic gene sets uniquely upregulated in P25 *Chd8*^*+/N2373K*^ females (whole brain) are no longer detectable at P56 and instead appear as downregulated gene sets in P56 *Chd8*^*+/N2373K*^ males and females (striatum), suggesting a loss of female-specific synaptic protective effects. Likewise, the mitochondrial and splicing gene sets downregulated at P25 in *Chd8*^*+/N2373K*^ females (whole brain) disappear by P56 but re-emerge as upregulated in P56 *Chd8* ^*N2373K/N2373K*^ females (whole brain), indicating that these pathways may contribute to the developmental shift from female protection to susceptibility.

Despite these robust findings, some limitations merit consideration. While our transcriptomic analyses have identified candidate biological functions and genes, they do not establish causal relationships between the diverse phenotypic changes observed, spanning behavior, brain structure, cerebral blood flow, neuronal firing, and synaptic transmission. Moreover, the development and suppression of sexually dimorphic phenotypes are complex processes influenced by developmental stage and brain region, complicating the establishment of definitive correlations across different domains.

In summary, our findings demonstrate that a homozygous Chd8 mutation in mice elicits robust ASD-like phenotypes while concurrently diminishing sexually dimorphic traits at multiple mechanistic levels. Transcriptomic analyses reveal that these effects are both developmentally and regionally specific, implicating critical biological processes related to synaptic function, mitochondrial activity, and splicing regulation in mediating these complex phenotypic outcomes.

## Methods

### Animals

The generation of the *Chd8*-mutant mice with the Asn2373LysfsX2 mutation in the genetic background of C57BL/6 J were described in a previous report [19]. These mice were back-crossed into the 129S1/SvlmJ strain by mating heterozygous B6J mice with wild-type 129S1/SvlmJ strain for at least five generations. The mice used in all experiments were F1 siblings produced by mating C57BL/6 J HT and 129/Sv HT mice, which were maintained independently, with the parental sex of each inbred line counterbalanced. All mice were maintained and handled according to the Requirements of Animal Research at KAIST. The animals were housed under a 13:00–01:00 dark/light cycle environment.

### PCR genotyping

For genotyping, the following primers were used. For1: CTT TAC CGG TGA GTT ACA TCA TCA, Rev1: CAG GCA AGC ACC TGG TGC ACA. To quantify mRNA levels, total brain RNAs were extracted from three pairs of WT and Chd8 + /N2373K male and female mice (P0), and cDNAs were synthesized using the TOPscript cDNA Synthesis Kit (Enzynomics, EZ005). SsoAdvanced SYBR Green Supremix (BIORAD, 1725260) and CFX96 Real-Time System were used for real-time PCR. The following primers were used. For2: GCG GAG CTG GAG ATG TGG TTA CAG, Rev2: TCT AGA GTT CGC TGG CTG TAC TGG T for CHD8. GAPDHF: TCA GCA ATG CAT CCT GCA CCA CC, GAPDHR: TGG CAG TGA TGG CAT GGA CTG TG for GAPDH and normalization. For sequencing validation of the knock-in (KI) mutation, PCR and gel extraction were performed on genomic DNA using the For1 and Rev1 primers.

### Immunoblot analysis

After immunoblotting, HRP-conjugated secondary antibody signals for Chd8 were detected by film exposure, while fluorescent secondary antibody signals were detected using the Odyssey Fc Dual Mode Imaging System. Signals were quantified using Image Studio Lite (Ver 4.0). The following antibodies were purchased: n-CHD8 (Bethyl, A301-224A), c-CHD8 (Bethyl, A301-225A), and β -actin (Sigma, A5316).

### Behavioral tests

For nocturnal mice, all behavioral tests were conducted during the night (light-off periods) using age- and sex-matched mice. A minimum rest period of 2 days was provided between tests to ensure recovery and reduce stress. All animals were randomly assigned to test days and order, counterbalanced across genotype and sex to minimize potential bias. We did not control for estrous cycle variability, as the cycles of group-housed females are generally synchronized.

### LABORAS

Each mouse was placed in an individual cage, and behaviors were recorded for 72 h, starting with the night cycle. Basal activities were recorded and automatically analyzed using the Laboratory Animal Behavior Observation Registration and Analysis System (LABORAS, Metris). Data from the 36-hour dark period were quantified and presented.

### Open-field test

An empty white acrylic box measuring 40 ×40 x 40 cm, referred to as an open-field box, was used to monitor mouse activity under 100 lux illumination. Mice were placed in the box for 60 min, and their activity was recorded. The videos were analyzed using the EthoVision XT program. The “distance moved” by the mice was used as a measure of their activity level, while the “time spent in the center” of the box was used to assess anxiety-like behavior.

### Repetitive behavior

Mice were individually placed in a home cage, a familiar environment, under 60 lux illumination. After a 10-minute habituation period, mouse activity was recorded for an additional 10 min. Self-grooming and digging behaviors were manually quantified during this recording period. For precise measurement of each behavior, two cameras were used to record from different angles.

### Light-dark box test

The light-dark box consisted of two connected chambers: a white, open-roof chamber (21 ×29 x 20 cm) and a closed black chamber (21 ×13 x 20 cm), with a small entrance allowing movement between the two. The light chamber was illuminated at 600 lux. Mice could freely move between the light and dark chambers, typically seeking the dark chamber when anxious in the bright environment. The time spent in the light chamber was measured as an indicator of anxiety-related behavior.

### Elevated plus-maze

The elevated plus-maze consists of two open arms and two closed arms, each measuring 5 ×30 cm. The closed arms have 30-cm-high walls, and the entire maze is elevated 50 cm above the floor. Mice were placed in the center of the maze and their movements were recorded for 8 min under 180 lux illumination. The time spent in each set of arms (open versus closed) was measured using the EthoVision XT program to assess anxiety-related behavior.

### Three-chamber test

Mice were first habituated in the center chamber for 10 min, followed by 10 min of exploration in all three chambers (Session 1). After Session 1, an age- and sex-matched Social 1 mouse (C57BL/6 J) was placed in a small container in the corner of one side chamber, while an object was placed in a container in the opposite side chamber. The mouse’s movements were then recorded for 10 min (Session 2). In Session 3, the object was replaced with another age- and sex-matched Social 2 mouse (C57BL/6 J), and the movements were recorded for another 10 min. Time spent in each chamber was analyzed using EthoVision XT 10.1, and sniffing time was manually quantified.

### Direct social-interaction test

For two consecutive days before the test, each mouse was habituated to an acrylic direct social interaction box for 10 min. On the test day, each mouse was placed in the box with a sex-matched wild-type (WT, C57BL/6 J) mouse, and their interactions were recorded for 10 min. The subject mouse’s behaviors, such as nose-to-nose sniffing, body contact, and nose-to-body sniffing, were manually quantified.

### Courtship ultrasonic vocalization (USV)

Ultrasonic vocalizations (USVs) were recorded using Avisoft Recorder software. An ultrasound microphone (Avisoft) was positioned 20 cm above the testing arena. Each mouse was placed in a home cage and habituated to the testing chamber for 5 min. Following habituation, an unfamiliar age-matched wild-type (WT, C57BL/6 J) female mouse was introduced, and the USVs were recorded for 5 min. The recorded USV calls were then transferred to Avisoft SASlab Pro for analysis.

### MRI: acquisitions

Five mice per group were used for the MRI studies (WTM, 36.2 ± 5.0 g; WTF, 26.5 ± 2.2 g; HTM 34.4 ± 3.4 g; HTF, 28.1 ± 2.6 g; HMM, 33.3 ± 1.9 g; and HMF, 25.9 ± 1.9 g). All MRI experiments were conducted on a Bruker Biospec 9.4 T/30-cm horizontal bore instrument with a 12.0-cm actively shielded insert, operating at a maximum gradient strength of 66 Gauss/cm and a rise time of 141 μs. The mouse brain was positioned near the isocenter of the magnet, using an 86 mm inner diameter quadrature birdcage coil for excitation and a 10-mm receiver surface coil on the mouse’s head. Magnetic field homogeneity was globally shimmed, followed by local optimization using the MAPSHIM protocol, targeting an ellipsoid volume covering the cerebrum (ParaVision 6.0.1, Bruker BioSpin). Single-shot GE-EPI sequences were acquired for total-vasculature perfusion mapping with parameters: TR/TE = 1000/11.6 ms, flip angle = 50°, receiver bandwidth = 300 kHz, spatial resolution = 156 μm × 156 μm × 500 μm, 20 interleaved slices, slice thickness = 500 μm, and 10 dummy scans. Physiological data, including heart rate, peripheral oxygen saturation, respiration, and end-tidal CO2, were continuously monitored using a multiparameter physiological monitoring system, with temperature maintained at 37° ± 0.5 °C using a warm water blanket.

### MRI: anesthetic regimes

Mice were initially anesthetized with 5% isoflurane (ISO) in a 1:4 mixture of oxygen and air for 3 to 4 min. The ISO concentration was then reduced to 2.5% for the MRI setup, and subsequently adjusted and maintained at 1.5% for the duration of the procedure. Data acquisition began 15 to 20 min after adjusting the ISO dose to ensure the anesthesia had stabilized. All experiments were completed within 1 h from the start of the first acquisition in each mouse. To ensure comparable depths of anesthesia across experimental groups, we measured baseline respiratory rates during imaging sessions. Baseline respiratory rates (mean ± SD) were as follows: WTM, 142.1 ± 20.4 BPM; WTF, 143.7 ± 15.9 BPM; HTM, 136.6 ± 8.0 BPM; HTF, 148.3 ± 16.5 BPM; HMM, 139.5 ± 3.9 BPM; and HMF, 144.2 ± 10.1 BPM. Respiratory rates were similar across groups, except for HTM.

### MRI: setup and stimulus designs

Two inhaled gas mixtures were utilized: one for control medical gas with anesthetics and the other for the hypoxic stimulus [53, 90]. These gases were connected to a breathing cone, with a transistor-transistor logic (TTL) signal synchronized with the MRI scanner to accurately switch between the two gases. A solenoid two-way pinch valve controlled the flow of medical gas to the animal, ensuring consistent gas pressure during experiments, while a three-channel programmable gas mixer (GSM-3 Gas Mixer, CWE Inc., Ardmore, PA) regulated the hypoxic gas stimulus. To efficiently remove exhaled and residual gas from the nose cone, a slightly negative pressure was maintained in the exhaust gas line. The normoxic baseline was set to 40% O2 balanced with N2 to ensure adequate oxygenation during anesthesia. The transient hypoxic stimulus, delivered without any inhaled anesthetics, used 100% N2. Each run, lasting 3 min and 20 s, consisted of 20 s of normoxia followed by a cycle of 5 s of hypoxia and 55 s of normoxia, repeated three times. For each anesthetic condition in each animal, five runs (totaling 15 trials of 5-second hypoxia) were conducted to gather data for total vasculature-sensitive GE-EPI. To ensure adequate BOLD sensitivity, we calculated the voxel-wise temporal SNR as the mean signal divided by the SD of the baseline. The whole-brain temporal SNR values (mean ± SD) were as follows: WTM, 23.3 ± 1.3; WTF, 24.3 ± 0.6; HTM, 23.9 ± 1.4; HTF, 23.2 ± 0.8; HMM, 23.8 ± 0.5; and HMF, 24.7 ± 0.6. No significant group differences were detected by ANOVA.

### MRI: Perfusion image analysis

All BOLD time series data for each mouse were analyzed using several tools and software packages, including the Analysis of Functional Neuroimages (AFNI) package [91], FMRIB Software Library (FSL), Advanced Normalization Tools (ANTs) [92], and custom MATLAB scripts (MathWorks). The preprocessing of individual EPI images involved the following steps: slice timing correction, image realignment, and linear detrending to remove signal drift.

Since anesthesia was used, head motion was expected to be minimal. Nonetheless, to ensure high stability in data collection, motion was quantified using frame-wise displacement across all EPI scans. The frame-wise dispalcements (mean ± SD) were as follows: WTM, 9.8 ± 4.5 μm; WTF, 8.3 ± 4.7 μm; HTM, 8.5 ± 3.4 μm; HTF, 8.1 ± 3.2 μm; HMM, 8.4 ± 3.8 μm; and HMF, 7.6 ± 2.8 μm. As expected under anesthesia, head motion was minimal, and no significant group differences were observed.

Cerebral blood volume (CBV) and cerebral blood flow (CBF) were quantified during the perfusion analysis, and group-averaged perfusion maps were generated within the mouse brain template space through a series of steps. First, T2-weighted anatomical images (78 μm × 78 μm × 500 μm) from all individual subjects were averaged using linear transformations to create a mouse brain template (anatomy template). Next, the Allen Mouse Brain Atlas and its labels were normalized to this mouse brain template using nonlinear transformations (Allen’s Atlas → anatomy template). Third, perfusion maps from individual EPI datasets (156 μm × 156 μm × 500 μm) were co-registered to the anatomy template using linear transformations (perfusion map → anatomy template). Finally, the perfusion maps aligned to the anatomy template were statistically analyzed based on the Allen’s Atlas space, integrating the steps (perfusion map → anatomy template ← Allen’s Atlas) [93].

The perfusion metrics were quantified using the dynamic susceptibility contrast (DSC) tracer kinetic theory [94–96], following the methods outlined in previous studies [53, 90]. Briefly, the EPI signal intensity change in each voxel was converted to the relaxation rate change ΔR2* using the formula for hypoxia-induced relative change: e^-TE*ΔR2∗^. The venous output function (VOF) and arterial input function (AIF) for the perfusion analysis were determined by calculating the area under the curve (AUC) within the first 12 s after stimulus onset and the peak intensity of the ΔR2*(t) signal [90], utilizing a vascular-atlas-based mask for VOF and AIF [97]. Subsequently, CBV was calculated as the ratio of the voxel’s AUC to the AUC of the AIF, corrected by the VOF. The tissue concentration response (Ct(t)), proportional to CBF, was defined as the convolution of the tissue response function R(t) [CBF × residue function R(t)] and the AIF, given by Ct(t) = CBF · AIF⨂R(t) [98]. The tissue impulse response function was obtained through deconvolution using the singular value decomposition (SVD) approach with a fixed threshold (20% cutoff) [53]. CBF was then determined as the maximum height of the tissue impulse response function.

In our whole-brain perfusion experiments using 20 slices at 0.5-mm thickness, the most anterior regions (e.g., olfactory bulb, frontal pole) and the most posterior regions (e.g., medulla and cerebellum) were only partially included. In addition, the ventral regions (olfactory ventral, retrohippocampal area, cortical subplate, etc.) exhibited distortion and signal loss in the EPI images. Therefore, these regions were excluded from the quantitative analyses shown in Fig. 3b and Fig. 4b.

To assess whether the measured CBV was influenced by total brain volume, we quantified intracranial volume covered by MRI (mean ± SD): WTM, 39.8 ± 0.6 mm³; WTF, 40.3 ± 0.9 mm³; HTM, 40.5 ± 1.2 mm³; HTF, 41.1 ± 0.6 mm³; HMM, 41.9 ± 0.8 mm³; and HMF, 42.1 ± 0.7 mm³. We then performed ANCOVA with brain volume and body weight as covariates and compared the results with those obtained from ANOVA (Supplementary Fig. 3e).

### Neuropixels: surgery, recordings, and analyses

A total of 52 mice were used for Neuropixels 1.0 recordings under urethane anesthesia (male WT: 9, male HT: 9, male HM: 9; female WT: 7, female HT: 7, female HM: 9; postnatal 9–16 weeks). An additional 12 mice were used for head-fixed awake Neuropixels 1.0 recordings (male WT: 3, male HM: 3; female WT: 3, female HM: 3; postnatal 9–16 weeks) to assess whether anesthetic state qualitatively altered genotype- and sex-dependent effects. For anesthetized in vivo recordings, mice were secured in a stereotaxic frame under urethane anesthesia (1.5 g/kg, i.p.). Body temperature was continuously monitored and maintained with a TC-1000 temperature controller (CWE), and the eyes were coated with Vaseline to prevent corneal drying. To minimize state-related variability, all animals received the same weight-adjusted urethane dose, recordings were initiated after a fixed post-induction interval, and recording durations were matched across animals. Respiratory rate and reflexes remained stable throughout, and a pinch-response test was performed at the start of each session to confirm a stable anesthetic depth. No supplemental doses were required, consistent with the use of urethane as a highly stable and reproducible anesthetic for long-duration recordings. For head-fixed awake recordings, mice underwent a craniotomy one week prior to the experiment. A custom head-plate was affixed to the skull using dental acrylic and titanium screws to ensure stable head fixation. A ground/reference screw was implanted over the nasal bone. Animals were habituated to the head-fixation apparatus for at least three days. On the recording day, mice were secured in the head-post clamp, and data were collected in a rig equipped with noise shielding and vibration isolation.

Neuronal activity was recorded across various brain regions, including cortical and thalamic areas. Two Neuropixels 1.0 probes were inserted bilaterally at coordinates AP 2.1 mm, ML ± 1.6 mm from bregma, and −4.5 mm from the brain surface. The probes were coated with the fluorescent dye DiI (D7757, Thermo Fisher Scientific) to localize the recording sites. The location of each probe channel was determined by examining post-processed brain tissue for each mouse using Allen-CCF (https://github.com/cortex-lab/allenCCF). Recordings used for analysis were conducted for 45 min following an hour of post-insertion stabilization. Simultaneous recordings were made from 384 sites on a single Neuropixels probe. Electrophysiology data were acquired using OpenEphys (https://open-ephys.org/gui). To obtain single-unit data, electrode signals were filtered between 300 Hz and 6000 Hz and sampled at 30,000 Hz. Spike sorting was performed using Kilosort1.5 (https://github.com/MouseLand/Kilosort/) [99], followed by manual curation to select isolated single units using Phy (https://github.com/cortex-lab/phy). Single units were selected after comprehensive inspection based on traditional measures such as inter-spike intervals (ISIs), autocorrelation, and waveform shape. The single-unit data were then analyzed to calculate firing rates using custom MATLAB code (www.mathworks.com).

Local field potential (LFP) data (sampling rate: 2000 Hz) were band-pass filtered between 0.1 and 200 Hz using a 3rd-order Butterworth filter, and a notch filter was applied to attenuate power line noise. The filtered LFP signals were analyzed using custom MATLAB scripts (www.mathworks.com). Fourier transformation was used to compute the power spectrum for each electrode channel. This was achieved by segmenting the data into 30-second bins and applying the Fast Fourier Transform (FFT) within each bin. The power spectra were then normalized by dividing the power at each frequency by the total power across all frequencies. The frequency bands of interest were defined as delta (0.1–4 Hz), theta (4–12 Hz), beta (13–30 Hz), low gamma (31–55 Hz), and high gamma (65–130 Hz). For each electrode channel, the power within these frequency bands was calculated by summing the normalized power spectra within the defined band limits. The power values for each frequency band and brain region were then log-transformed to facilitate analysis. Fold changes were calculated by first averaging the log-transformed power values for each group, and then dividing the group average by the average of either the male WT or the female WT group. This approach allowed for the comparison of relative changes in power across different frequency bands and between experimental groups. At the end of the experiment, brains were extracted, then sectioned (70 µm slices). The probe tracks were mapped onto brain atlas coordinates to verify targeting accuracy and recording depth using SHARP-Track ss(https://doi.org/10.1101/447995). High-resolution coronal sections were registered to the Allen Mouse Brain Atlas, and the Neuropixels shank was manually aligned to the reconstructed track to determine the entry point, insertion angle, and depth profile. Each electrode site and each sorted unit was assigned an anatomical depth and brain region based on this reconstruction, and only units and LFP channels located within brain regions that were consistently sampled across all animals were included in between-group comparisons, ensuring that genotype- or sex-dependent differences could not be attributed to differential laminar or regional sampling.

### Electrophysiology

For adult hippocampus recording, acute sagittal brain slices were obtained by anesthetizing P56 mice with isoflurane and extracting the brain after perfusion with NMDG buffer (pH 7.3 ~ 7.4 and 300 ~ 310 mOsm/kg) containing NMDG (100 mM), N-acetylcysteine (NAC, 12 mM), NaHCO_3_ (30 mM), HEPES (20 mM), Glucose (25 mM), Thiourea (2 mM), Na-Ascorbate (5 mM), Na-pyruvate (3 mM), KCl (2.5 mM), NaH_2_PO_4_ (1.25 mM), CaCl_2_ (0.5 mM), and MgSO_4_ (10 mM). The extracted brains were sliced by vibratome (VT1200s, Leica) with 300 μm thickness and were incubated in 32 °C recovery chamber for 11 min and then transferred to RT with HEPES aCSF for 1 h, which consists of NaCl (92 mM), NAC (12 mM), NaHCO_3_ (30 mM), HEPES (20 mM), glucose (25 mM), thiourea (2 mM), Na-ascorbate (5 mM), Na-pyruvate (3 mM), KCl (2.5 mM), NaH_2_PO_4_ (1.25 mM), MgCl_2_ (1.3 mM), and CaCl_2_ (2.5 mM).

The whole-cell voltage clamp was conducted under circulation of aCSF consist of NaCl (125 mM), NaHCO_3_ (25 mM), KCl (2.5 mM), NaH_2_PO_4_ (1.25 mM), D-glucose (10 mM), MgCl_2_ (1.3 mM), and CaCl_2_ (2.5 mM). Thin-walled borosilicate capillaries (30-0065, Harvard Apparatus) were used to make pipettes with resistance 2.3 ~ 3.5MΩ via a two-step vertical puller (PC-10, Narishige). For sEPSC and mEPSC recordings, pipettes were filled with an internal solution composed of, in mM: 117 CsMeSO4, 10 EGTA, 8 NaCl, 10 TEACl, 10 HEPES, 4 Mg-ATP, 0.3 Na-GTP, 5 QX-314. For sIPSC and mIPSC recordings, the internal solution contained, in mM: 115 CsCl, 10 EGTA, 8 NaCl, 10 TEACl, 10 HEPES, 4 Mg-ATP, 0.3 Na-GTP, 5 QX-314. All internal solutions were titrated to pH 7.35 and adjusted to an osmolarity of 285 mOsm. For sEPSC experiments, 60 μM picrotoxin (Sigma) was added to the aCSF. For mEPSC experiments, 60 μM picrotoxin and 0.5 μM tetrodotoxin (Tocris) were added. For sIPSC experiments, 10 μM NBQX (Tocris), 50 μM D-AP5 (Tocris) were added. For mIPSC experiments 10 μM NBQX (Tocris), 50 μM D-AP5 (Tocris), 0.5 μM tetrodotoxin (Tocris) were added. For cell property experiments, 10 μM NBQX (Tocris), 50 μM D-AP5 (Tocris), and 60 μM picrotoxin.

We used only the neurons in specific property ranges (access resistance/Ra < 20 MΩ, membrane resistance/Rm > 100 MΩ, cell capacitance/Cm > 100 pF) for recordings and analyses. The neurons within these ranges did not display genotype/sex differences. Signals were filtered at 2 kHz and digitized at 10 kHz using the Multiclamp 700B Amplifier (Molecular Devices) and the Digidata 1550 Digitizer (Molecular Devices). Cells were approached with an internal solution-filled pipette to establish a giga seal, followed by gentle suction to rupture the membrane and establish whole-cell configuration, with cells maintained at –70 mV. After stabilizing the voltage-clamped cells (~3 min post-rupture), recordings were obtained. Access resistance was continuously monitored throughout the stabilization period and immediately before and after data acquisition. The acquired data were analyzed using Clampfit 10 software (Molecular Devices).

### RNA-Seq analyses

Whole brains from P0, P25, and P56 mice, along with dissected prefrontal cortex, striatum, and hippocampus from P56 mice, were preserved in RNAlater solution (Ambion) to stabilize RNA. RNA extraction, library preparation, cluster generation, and sequencing were performed by Macrogen. Sequencing was conducted on an Illumina HiSeq 4000 platform, achieving an average read depth of 70–90 million paired-end reads (2×101 bp). Image analysis and base calling were performed using Illumina Real-Time Analysis (RTA) software, and raw BCL files were converted to FASTQ format using the Illumina bcl2fastq package.

The dataset comprised 180 samples across two experimental designs. For developmental analysis, 90 whole-brain samples were collected from P0, P25, and P56 mice, spanning both sexes and three genotypes (wild-type, heterozygous, homozygous). Sequencing for each age group was conducted in a separate batch (30 samples per batch), resulting in complete confounding between age and batch. For tissue-specific analysis at P56, 90 additional samples were collected from the prefrontal cortex, hippocampus, and striatum, again including both sexes and genotypes. Each tissue type was sequenced in its own batch, introducing confounding between tissue and batch. To account for batch confounding, differential gene expression (DGE) analyses were performed independently within each group (i.e., per age or per tissue type) using the DESeq2 package. Within each group, genotype and sex were modeled as factors, and appropriate covariates were included where applicable.

Transcript abundance was quantified using Salmon (v1.1.0) in quasi-mapping mode with GC bias correction, aligning to the *Mus musculus* reference transcriptome (GRCm38). Gene-level abundance estimates were imported into R (v3.5.3) using the tximport package. DGE analysis was conducted using DESeq2 (v1.30.1), which normalizes read counts by estimating size factors and models the data with a negative binomial distribution. Significance was determined using the Benjamini–Hochberg procedure to correct for multiple testing, with genes considered differentially expressed at adjusted *p* < 0.05.

Gene Ontology (GO) enrichment analysis was performed using DAVID (v6.8). Mouse gene symbols were converted to human orthologs using the Mouse Genome Informatics (MGI) database. Additionally, Gene Set Enrichment Analysis (GSEA) was carried out with the GSEAPreranked module from the GSEA software (MSigDB v7.5.1). Ranked gene lists comparing wild-type and *Chd8* heterozygous mice were tested against predefined gene sets using 1,000 permutations and a classic enrichment score. Gene sets with a false discovery rate (FDR) < 0.05 were considered significantly enriched.

### Statistical analysis

All behavioral datasets were analyzed using two-way ANOVA with genotype and sex as fixed factors. Primary endpoints were pre-specified as: (1) locomotor activity (distance in LABORAS and open field), (2) anxiety-like behavior (open-field center time, light–dark light-chamber time, EPM closed-arm time), (3) self-grooming (LABORAS and home-cage assays), and (4) social behavior (three-chamber preference indices and direct-interaction time); secondary measures (e.g., sniffing times, USV parameters) were not included in multiplicity correction. When the genotype × sex interaction was significant (*p* < 0.05), we performed pre-planned simple-effects tests between genotypes within each sex and adjusted p values using the Holm-Sidak step-down procedure (FWER = 0.05 within each primary endpoint); when no interaction but a genotype main effect was present, we used one-way ANOVA or t tests within sex. Supplementary Table 1 summarizes, for each endpoint and sex, group means, standard deviations, 95% confidence intervals, absolute mean differences (HT–WT and HM–WT), and Cohen’s d for WT–HT and WT–HM contrasts; per-sex sample sizes are given there and in all figure legends. Outliers were identified using Grubbs’ test (α = 0.05) and removed only when statistically justified, and all analyses were performed in GraphPad Prism.

## Supplementary information

Supplementary information

Supplementary Table 1

Supplementary Table 2

Supplementary Table 3

Supplementary Table 4

## Data Availability

The RNA-Seq datasets available under GEO accession numbers GSE275918 and GSE275742 provide gene expression data from the whole brain at developmental stages P0, P25, and P56, and from specific brain regions (cortex, hippocampus, and striatum) at P56, respectively. These datasets are also combined under the SuperSeries GSE275960.
