# Supplementary material for: Homozygous CHD8 mutation intensifies ASD phenotypes and attenuates sex differences
Source: Mol Psychiatry. 2026 May 9;31(10):5629–45. doi: 10.1038/s41380-026-03646-9 (PMC13569427; doi:10.1038/s41380-026-03646-9)
Supplement: Supplementary file 1 [file 41380_2026_3646_MOESM1_ESM.docx]

Homozygous CHD8 mutation intensifies ASD phenotypes and attenuates sex differences

Jinkyeong Kim^1,*^, Seungjoon Lee^2,*^, Eunkyu Hwang^3,*^, Hwajin Jung^2^, Chanhee Lee^4,5^, Sang-Han Choi^4,5^, Sooyeon Lee^1^, Seongbin Kim^1^, Heera Moon^1^, Jisoo Kim^1^, Gina Lee^1^, Yong Gyu Kim^2^, Soogeun Shin^3^, Hyojin Kang^6^, Se Jin Kim^7^, Heon Yung Gee^7^, Seong-Gi Kim^4,5^, Eunee Lee^2,3,#^, and Eunjoon Kim^1,2,#^

^1^Department of Biological Sciences, Korea Advanced Institute of Science and Technology (KAIST), Daejeon, Korea; ^2^Center for Synaptic Brain Dysfunctions, Institute for Basic Science (IBS), Daejeon, Korea; ^3^Department of Anatomy, Graduate School of Medical Science, Yonsei University College of Medicine, Brain Korea 21 Project, Seoul, Korea; ^4^Center for Neuroscience Imaging Research, Institute for Basic Science (IBS), Suwon, Korea; ^5^Department of Biomedical Engineering, Sungkyunkwan University, Suwon, Korea; ^6^Division of National Supercomputing, Korea Institute of Science and Technology Information (KISTI), Daejeon, Korea; ^7^Department of Pharmacology, Graduate School of Medical Science, Yonsei University College of Medicine, Brain Korea 21 Project, Seoul, Korea; *These authors contributed equally; ^#^Co-corresponding authors

**Supplementary Figures and Legends**

**Supplementary Figure 1. Decreased levels of CHD8 proteins in various brain regions.**

(a–f) Decreased levels of CHD8 proteins in different brain regions (cortex, hippocampus, and striatum) of *Chd8^+/N2373K^* and *Chd8^N2373K/N2373K^* mice (P6; males and females mixed; indicated by blue and red colors, respectively), as determined by immunoblot analyses using total lysates and N- and C-terminal CHD8 antibodies (target regions indicated in Fig. 1d). (n = 2 [M-WT], 2 [M-HT], 1 [M-HM], 3 [F-WT], 3 [F-HT], 3 [F-HM]).

**Supplementary Figure 2. Sexually dimorphic behavioral deficits in *Chd8^N2373K/N2373K^* mice analyzed with outliers included.**

(a) Schema of behavioral experiments for male and female *Chd8^+/N2373K^* and *Chd8^N2373K/N2373K^* mice (3–4.5 months).

(b) Locomotor activity in the Laboras test (familiar environment) as shown by distance moved by male and female *Chd8^+/N2373K^* and *Chd8^N2373K/N2373K^* mice. Full details of the statistical analyses are provided in **Supplementary Table 1**. (n = 17 mice [M/male-WT/wild-type], 14 [M-HT/heterozygote; 17 [M-HM/homozygote], 15 [F/female-WT], 12 [F-HT], 13 [F-HM], two-way ANOVA).

(c) Locomotor activity in the open-field test (novel environment) as shown by distance moved. (n = 17 [M-WT], 13 [M-HT], 13 [M-HM], 18 [F-WT], 13 [F-HT], 17 [F-HM]), two-way ANOVA, one-way ANOVA within sex [indicated by blue asterisks]).

(d) Time spent in center during the open-field test, as a measure of anxiety-like behavior. (n = 17 [M-WT], 13 [M-HT], 13 [M-HM], 18 [F-WT], 13 [F-HT], 17 [F-HM]), two-way ANOVA, one-way ANOVA within sex).

(e) Light-dark test for anxiety-like behavior. (n = 18 [M-WT], 13 [M-HT], 14 [M-HM], 18 [F-WT], 13 [F-HT], 17 [F-HM], two-way ANOVA, one-way ANOVA within sex).

(f) Elevated plus-maze test for anxiety-like behavior. (n = 18 [M-WT], 13 [M-HT], 14 [M-HM], 18 [F-WT], 13 [F-HT], 17 [F-HM], two-way ANOVA).

(g) Self-grooming in Laboras cages. (n = 17 [M-WT], 14 [M-HT], 17 [M-HM], 15 [F-WT], 12 [F-HT], 13 [F-HM], two-way ANOVA, one-way ANOVA within sex).

(h) Self-grooming in home cages. (n = 18 [M-WT], 13 [M-HT]; 14 [M-HM], 18 [F-WT], 13 [F-HT], 17 [F-HM], two-way ANOVA).

(i) Courtship ultrasonic vocalizations (USVs). (n = 18 [M-WT], 13 [M-HT], 14 [M-HM], one-way ANOVA).

(j) Direct social-interaction test. (n = 18 [M-WT], 12 [M-HT], 14 [M-HM], 18 [F-WT], 13 [F-HT], 17 [F-HM], two-way ANOVA, one-way ANOVA within sex).

(k) Three-chamber social-interaction tests. S1, social target; O, object; S2, novel social target. Preference index: S1–O/S1+O or S2–S1/S2+S1. (n = 17 [M-WT], 13 [M-HT], 13 [M-HM], 18 [F-WT], 13 [F-HT], 17 [F-HM], two-way ANOVA, Student’s t-test [S1 vs. O or S1 vs. S2], one-way ANOVA within sex).

(l) Summary of the behavioral results from *Chd8^+/N2373K^* and *Chd8^N2373K/N2373K^* males and females. Red/blue colors indicate fold changes (increases/decreases) in HT/HM mice relative to WT mice, and stars indicate significant differences between HT/HM and WT mice (one-way ANOVA followed by Dunnett’s test).

Significance on two-way ANOVA is indicated as * (<0.05), ** (<0.01), *** (<0.001), or ns (not significant).

**Supplementary Figure 3. Cerebral blood flows in *Chd8^+/N2373K^* and *Chd8^N2373K/N2373K^* males and females.**

(a) Cerebral blood flows (CBFs) in different brain regions of *Chd8^+/N2373K^* and *Chd8^N2373K/N2373K^* male and female mice (~4–5 months), measured by fMRI responding to 5-sec nitrogen stimulus under anesthesia (isoflurane). Red/blue colors indicate fold changes (increases/decreases) in HT/HM mice relative to WT mice. (n = 5 mice [M-WT], 5 [M-HT; 5 [M-HM], 5 [F-WT], 5 [F-HT], 5 [F-HM]).

(b) Color-coded display of altered cerebral blood flows in the different brain regions shown in (a). Stars indicate significant differences between HT/HM and WT mice within males/females (one-way ANOVA with Dunnett’s test). The whole brain value represents the average flow rate across all the indicated brain regions.

Significance is indicated as * (<0.05), ** (<0.01), *** (<0.001), or ns (not significant).

**Supplementary Figure 4. BOLD dynamics induced by transient hypoxia and the relationship between CBV and regional brain volume.**
**(a)** Mean normalized time courses in the hippocampus (HIP) and a pons-behavioral (P-sat) region evoked by brief 100% N₂ hypoxic challenges, measured with BOLD–DSC MRI. These two regions were selected because they showed significant CBV differences between WT and HM mice. These two regions were chosen due to significant differences in CBV between WT and HM. Across genotypes and sexes, vascular dynamics, including onset, peak timing, and recovery, were highly similar. Group differences in CBV were mainly reflected in the **magnitude** of the peak response rather than temporal profile of the response. A subtle sex difference is evident in the recovery phase, particularly in P-sat, with females showing slightly slower return to baseline.
**(b)** Summary of p values for correlations between regional CBV and regional brain volume across all ROIs. No region shows a significant CBV–volume correlation after correction, suggesting that global anatomy–function coupling is weak at this level of analysis.
**(c and d)** Example scatter plots illustrating the strongest **uncorrected** negative associations between CBV and regional volume in the prelimbic (PL) and retrosplenial (RSP) cortices, respectively. Linear regression lines and statistics (p value, R², and slope) are shown, indicating that larger regional volumes tend to be associated with lower CBV in these areas, although these effects do not survive multiple-comparisons correction.

**(e)** **ANCOVA results testing whether group differences in CBV remain after covarying for total brain volume and body weight (model: CBV ~ Group + Brain Volume + Body Weight), shown together with p values from a one-way ANOVA on raw CBV (model: CBV ~ Group) for comparison.** Bars indicate p values for the group term across ROIs, with the FDR threshold marked. **The broadly similar pattern of p values between ANOVA and ANCOVA indicates that adjusting for total brain volume and body weight does not substantially change the group effects on CBV, suggesting that the observed CBV differences are not trivially explained by these global covariates.**

**Supplementary Figure 5. Brain rhythms** **in multiple brain regions of *Chd8^+/N2373K^* and *Chd8^N2373K/N2373K^* males and females under anesthesia**.

(a–d) Local rhythms in multiple brain regions (cortex, CA1, DG, and thalamus) of *Chd8^+/N2373K^* and *Chd8^N2373K/N2373K^* males and females (9–16 weeks; urethane anesthesia), as measured by Neuropixels recordings of local field potentials and analysis of their levels in different frequency ranges (delta, theta, beta, low gamma, and high gamma). (n = 9 mice [M-WT], 8 [M-HT], 8 [M-HM], 5 [F-WT], 6 [F-HT], and 8 [F-HM], two-way ANOVA, one-way ANOVA [within-sex comparison of WT, HT, and HM]).

Significance is indicated as * (<0.05), ** (<0.01), *** (<0.001), or ns (not significant).

**Supplementary Figure 6. Brain rhythms** **in multiple brain regions of *Chd8^+/N2373K^* and *Chd8^N2373K/N2373K^* males and females in awake states**.

(a–d) Local rhythms in multiple brain regions (cortex, CA1, DG, and thalamus) of *Chd8^+/N2373K^* and *Chd8^N2373K/N2373K^* males and females (9–16 weeks; awake state), as measured by Neuropixels recordings of local field potentials and analysis of their levels in different frequency ranges (delta, theta, beta, low gamma, and high gamma). (Cortex, n = 4 hemispheres from 3 mice [M-WT], 3, 3 [M-HM], 5, 4 [F-WT], 4, 3 [F-HM]; CA1, 4, 3 [M-WT], 3, 3 [M-HM], 7, 4 [F-WT], 4, 3 [F-HM]; DG, 4, 3 [M-WT], 3, 3 [M-HM], 6, 4 [F-WT], 4, 3 [F-HM]; Th, 4, 3 [M-WT], 3, 3 [M-HM], 6, 4 [F-WT], 4, 3 [F-HM], two-way ANOVA, unpaired t-test [within-sex comparison of WT and HM]).

Significance is indicated as * (<0.05), ** (<0.01), *** (<0.001), or ns (not significant).

**Supplementary Figure 7. Synaptic transmission in hippocampal neurons from *Chd8^+/N2373K^* and *Chd8^N2373K/N2373K^* males and females**.

(a) mEPSC frequency and amplitudes in hippocampal CA1 pyramidal neurons in *Chd8^+/N2373K^* and *Chd8^N2373K/N2373K^* males and females (~P56–70). (n = 17 neurons from 6 mice [male-WT], 25, 6 [male-HT], 22, 6 [male-HM], 27, 6 [female-WT], 20, 6 [female-HT], 19, 5 [female-HM], two-way ANOVA with Holm-Sidak’s test).

(b) mIPSC frequency and amplitudes in hippocampal CA1 pyramidal neurons in *Chd8^+/N2373K^* and *Chd8^N2373K/N2373K^* males and females (~P56–70). (n = 20, 6 [male-WT], 18, 6 [male-HT], 23, 6 [male-HM], 24, 6 [female-WT], 22, 6 [female-HT], 26, 6 [female-HM], two-way ANOVA with Holm-Sidak’s test).

(c) sEPSC frequency and amplitudes in hippocampal CA1 pyramidal neurons in *Chd8^+/N2373K^* and *Chd8^N2373K/N2373K^* males and females (~P56–70). (n = 18, 7 [male-WT], 17, 6 [male-HT], 18, 6 [male-HM], 20, 6 [female-WT], 20, 6 [female-HT], 22, 6 [female-HM], two-way ANOVA with Holm-Sidak’s test).

(d) sIPSC frequency and amplitudes in hippocampal CA1 pyramidal neurons in *Chd8^+/N2373K^* and *Chd8^N2373K/N2373K^* males and females (~P56–70). (n = 20, 6 [male-WT], 18, 6 [male-HT], 14, 6 [male-HM], 18, 6 [female-WT], 20, 6 [female-HT], 16, 6 [female-HM], two-way ANOVA with Holm-Sidak’s test).

Significance is indicated as * (<0.05), ** (<0.01), *** (<0.001), or ns (not significant).

**Supplementary Figure 8. Synaptic transmission in prefrontal and striatal neurons from *Chd8^+/N2373K^* and *Chd8^N2373K/N2373K^* males and females**.

(a) sEPSC frequency and amplitudes in mPFC layer 2 pyramidal neurons from *Chd8^+/N2373K^* and *Chd8^N2373K/N2373K^* males and females (~P56–70). (n = 17 neurons from 3 mice [male-WT], 14, 3 [male-HM], 16, 4 [female-WT], 14, 4 [female-HM], two-way ANOVA).

(b) sIPSC frequency and amplitudes in mPFC layer 2 pyramidal neurons from *Chd8^+/N2373K^* and *Chd8^N2373K/N2373K^* males and females (~P56–70). (n =17, 3 [male-WT], 18, 3 [male-HM], 17, 3 [female-WT], 22, 4 [female-HM], two-way ANOVA).

(c) sEPSC frequency and amplitudes in mPFC layer 5 pyramidal neurons from *Chd8^+/N2373K^* and *Chd8^N2373K/N2373K^* males and females (~P56–70). (n = 18, 4 [male-WT], 16, 3 [male-HM], 12, 4 [female-WT], 12, 4 [female-HM], two-way ANOVA).

(d) sIPSC frequency and amplitudes in mPFC layer 5 pyramidal neurons from *Chd8^+/N2373K^* and *Chd8^N2373K/N2373K^* males and females (~P56–70). (n = 15, 3 [male-WT], 18, 3 [male-HM], 16, 3 [female-WT], 21, 4 [female-HM], two-way ANOVA with Holm-Sidak’s test).

(e) sEPSC frequency and amplitudes in dorsal striatal neurons from *Chd8^+/N2373K^* and *Chd8^N2373K/N2373K^* males and females (~P56–70). (n = 19, 6 [male-WT], 16, 6 [male-HM], 20, 6 [female-WT], 17, 6 [female-HM], two-way ANOVA).

(f) sIPSC frequency and amplitudes in dorsal striatal neurons from *Chd8^+/N2373K^* and *Chd8^N2373K/N2373K^* males and females (~P56–70). (n = 21, 6 [male-WT], 21, 6 [male-HM], 21, 6 [female-WT], 21, 6 [female-HM], two-way ANOVA).

Significance is indicated as * (<0.05), ** (<0.01), *** (<0.001), or ns (not significant).

**Supplementary Figure 9.** **Gene-set clustering derived from the GSEA results of the transcriptomic data from whole brains (P0, P25, and P56) of *Chd8^+/N2373K^* and *Chd8^N2373K/N2373K^* males and females.**

(a) The analysis focused on gene sets related to the cellular component domain of the Gene Ontology (GO) database. Gene-set clusters were generated using the Enrichment Cytoscape App. Gene sets that exhibit similar patterns in both male and female mutants are highlighted in bold and green. (n = 5 mice for WT/HT/HM-male/female-P0/P25/P56, FDR < 0.05).

**Supplementary Figure 10.** **Gene-set clustering derived from the GSEA results of the transcriptomic data from whole brains (P0, P25, and P56) of *Chd8^+/N2373K^* and *Chd8^N2373K/N2373K^* males and females.**

(a) The analysis focused on gene sets related to the biological process domain of the Gene Ontology (GO) database. Gene-set clusters were generated using the Enrichment CytoscapeApp. Gene sets that exhibit similar patterns in both male and female mutants are highlighted in bold and green. (n = 5 mice for WT/HT/HM-male/female-P0/P25/P56, FDR < 0.05).

**Supplementary Figure 11.** **Gene-set clustering derived from the GSEA results of the transcriptomic data from three distinct brain regions (Hp, Ctx, and Str; P56)** **of *Chd8^+/N2373K^* and *Chd8^N2373K/N2373K^* males and females.**

(a) The analysis focused on gene sets related to the cellular component domain of the Gene Ontology (GO) database. Gene-set clusters were generated using the Enrichment Cytoscape App. Gene sets that exhibit similar patterns in both male and female mutants are highlighted in bold and green. Hp, hippocampus; Ctx, cortex; Str, striatum. (n = 5 mice for WT/HT-male/female-Hp/Ctx/Str, FDR < 0.05).

**Supplementary Figure 12.** **Gene-set clustering derived from the GSEA results of the transcriptomic data from three distinct brain regions (Hp, Ctx, and Str; P56)** **of *Chd8^+/N2373K^* and *Chd8^N2373K/N2373K^* males and females.**

(a) The analysis focused on gene sets related to the biological process domain of the Gene Ontology (GO) database. Gene-set clusters were generated using the Enrichment Cytoscape App. Gene sets that exhibit similar patterns in both male and female mutants are highlighted in bold and green. Hp, hippocampus; Ctx, cortex; Str, striatum. (n = 5 mice for WT/HT-male/female-Hp/Ctx/Str, FDR < 0.05).

**Supplementary Figure 13. Leading-edge analysis for the mitochondria and splicing-related gene sets from *Chd8^+/N2373K^* females (P25; whole brain) and *Chd8^N2373K/N2373K^* females (P56; whole brain).**

(a) Leading-edge analysis results for the splicing-related gene sets from *Chd8^+/N2373K^* females (P25; whole brain). Genes common to this analysis and that in panel (c) are highlighted in green, while those with stronger fold changes and appearing across multiple gene sets are emphasized in bold.

(b) Leading-edge analysis results for the mitochondria-related gene sets from *Chd8^+/N2373K^* females (P25; whole brain). Genes common to this analysis and that in panel (d) are highlighted in green, while those with stronger fold changes and appearing across multiple gene sets are emphasized in bold.

(c) Leading-edge analysis results for the splicing-related gene sets upregulated in *Chd8^N2373K/N2373K^* females (P56; whole brain).

(d) Leading-edge analysis results for the mitochondria-related gene sets upregulated in *Chd8^N2373K/N2373K^* females (P56; whole brain).

**Supplementary Figure 14. Leading-edge analysis for the synapse-related gene sets from *Chd8^+/N2373K^* females (P25; whole brain) and *Chd8^N2373K/N2373K^* males and females (P56; cortex).**

(a) Leading-edge analysis results for the synapse-related gene sets from *Chd8^+/N2373K^* females (P25; whole brain). Genes common to this analysis and that in panel (b) are highlighted in green, while those with stronger fold changes and appearing across multiple gene sets are emphasized in bold.

(b) Leading-edge analysis results for the synapse-related gene sets shared between *Chd8^N2373K/N2373K^* males and females (P56; striatum); gene sets from the female were used to identify leading-edge genes.

**Supplementary Tables**

**Supplementary Table 1. Statistical details.**

**Supplementary Table 2. Raw RNA-Seq data.**

**Supplementary Table 3. List of DEGs.**

**Supplementary Table 4. GSEA results.**
